# Inspired by Nature—Functional Analogues of Molybdenum and Tungsten-Dependent Oxidoreductases

**DOI:** 10.3390/molecules27123695

**Published:** 2022-06-08

**Authors:** Sebastian Pätsch, Jevy V. Correia, Benedict J. Elvers, Mareile Steuer, Carola Schulzke

**Affiliations:** Institut für Biochemie, Universität Greifswald, Felix-Hausdorff-Str. 4, 17489 Greifswald, Germany; paetschs@uni-greifswald.de (S.P.); correiaj@uni-greifswald.de (J.V.C.); be123028@uni-greifswald.de (B.J.E.); mareile.steuer@stud.uni-greifswald.de (M.S.)

**Keywords:** oxidoreductases, dithiolene, OAT, functional models, hydrogen evolution, acetylene hydratase

## Abstract

Throughout the previous ten years many scientists took inspiration from natural molybdenum and tungsten-dependent oxidoreductases to build *functional* active site analogues. These studies not only led to an ever more detailed mechanistic understanding of the biological template, but also paved the way to atypical selectivity and activity, such as catalytic hydrogen evolution. This review is aimed at representing the last decade’s progress in the research of and with molybdenum and tungsten functional model compounds. The portrayed systems, organized according to their ability to facilitate typical and artificial enzyme reactions, comprise complexes with *non-innocent* dithiolene ligands, resembling molybdopterin, as well as entirely non-natural nitrogen, oxygen, and/or sulfur bearing chelating donor ligands. All model compounds receive individual attention, highlighting the specific novelty that each provides for our understanding of the enzymatic mechanisms, such as oxygen atom transfer and proton-coupled electron transfer, or that each presents for exploiting new and useful catalytic capability. Overall, a shift in the application of these model compounds towards uncommon reactions is noted, the latter are comprehensively discussed.

## 1. Introduction

**Figure 1:**
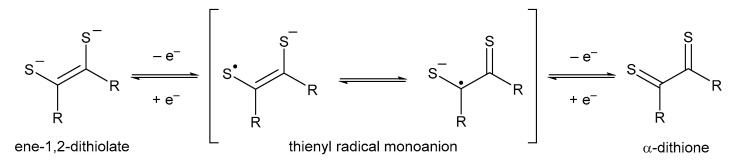
Potential redox activity (and intramolecular electron transfer) of the dithiolene ligand system constituting the foundation of its *non-innocent* nature.

The mononuclear molybdenum and tungsten-dependent oxidoreductases constitute a diverse and, beyond doubt, fascinating class of enzymes. With a few exceptions, they catalyze the overall simple but mechanistically complex oxygen atom transfer (OAT) reaction, by which formally a single oxygen atom (O^0^) is transferred to or from a suitable substrate. In general, these so-called oxotransferases are ubiquitously found in almost any cellular life form and play a key role in energy production, as well as the metabolism of carbon, sulfur, and nitrogen [1]. A special ligand system, molybdopterin (MPT), which contains a tricyclic pyranopterin moiety and a chelating ene-1,2-dithiolate coordination site, constitutes an important structural aspect of their respective active sites [2]. Its chelating ene-1,2-dithiolate, or shorter ‘dithiolene’ moiety, is a prime example for a *non-innocent* ligand with the ability to modulate the electron density of the coordinated complex center, because it is inherently susceptible to redox transitions (Figure 1). Since this intriguing ability is not the focus of this review, we provide no further details about the versatile history and chemistry of metal dithiolene complexes unless they are imminently relevant for a specific section. Respective comprehensive and very useful overviews have been published previously [2,3,4]. Due to the dithiolene’s *non-innocence*, all given oxidation states of the central metal ions in their complexes should be regarded as being ‘formal’ throughout this review article, including those indicated in the figures.

**Figure 2:**
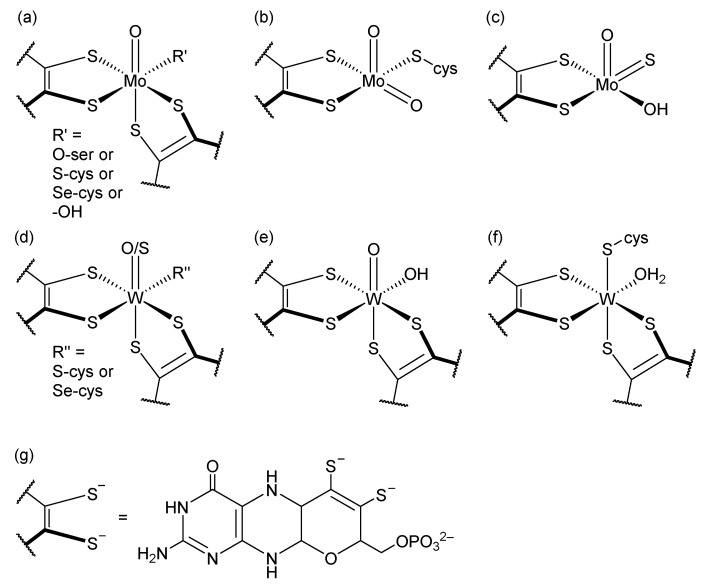
Classification of mononuclear molybdenum and tungsten-dependent oxidoreductases enzymes into families according to Hille (all shown as fully oxidized metals) and the chemical structure of molybdopterin: (**a**) DMSO reductase family (**b**) sulfite-oxidase family (**c**) xanthine oxidase family (**d**) formate dehydrogenase family (**e**) aldehyde ferredoxin oxidoreductase family (**f**) acetylene dehydratase (**g**) molybdopterin (MPT, reduced form) [5,6,7].

The active sites of different molybdenum and tungsten-dependent enzymes are not entirely uniform. They vary in the number of MPT ligands, in the presence or absence of a covalent/coordinative bond with the enzyme’s peptide, and in the nature of the one-atom ligands. This led Hille to classify them into different enzyme families (Figure 2) [5,6].

Model compounds with a close structural resemblance to the active sites bear, in consequence, one or two dithiolene ligands [8]. However, the enzymatic functions can also be mimicked by employing non-dithiolene ligand systems, rendering those complexes similarly important. Even more, such model complexes can provide deeper insights into structure function relationships by the absence of the non-innocent dithiolene. An example in this respect is the investigation of the coordination geometry of a hexacoordinated active site or model, respectively, that could be either trigonal-prismatic or octahedral and which depends on the employed ligands [9].

An enormous number of distinct bioinspired synthetic analogues were investigated with regard to their OAT reactivity. Among those examined were different oxygen atom donor/acceptor substrates, varied solvent systems, and the type of the co-ligand aside from variations in general complex composition. In some cases, one specific model system was tested as a catalyst for natural and/or unnatural reactivity. Unexpected catalytic and non-catalytic reaction pathways were observed pointing out the versatile functionality associated with the coordination chemistry of molybdenum and tungsten.

This review is showcasing the functional model chemistry for molybdenum-dependent oxidoreductase enzymes from approximately the last ten years (2011–2021). All respective activities prior to this decade have already been most comprehensively reviewed in various articles in a special issue of Coordination Chemistry Reviews from 2011 which was specifically dedicated to these enzymes and covers biological and chemical aspects, and in various further valuable (review) articles [1,2,8,9,10,11]. In the following, complexes are taken into consideration which are able to promote substrate transformations as they are observed for the natural enzymes—whether they bear dithiolene ligands as models for the biological active site ligand molybdopterin or not—and such complexes with a structural resemblance to these active sites with unusual, unexpected, or even unprecedented reactivities.

## 2. Functional Analogues Showing Natural Reactivity

**Figure 3:**
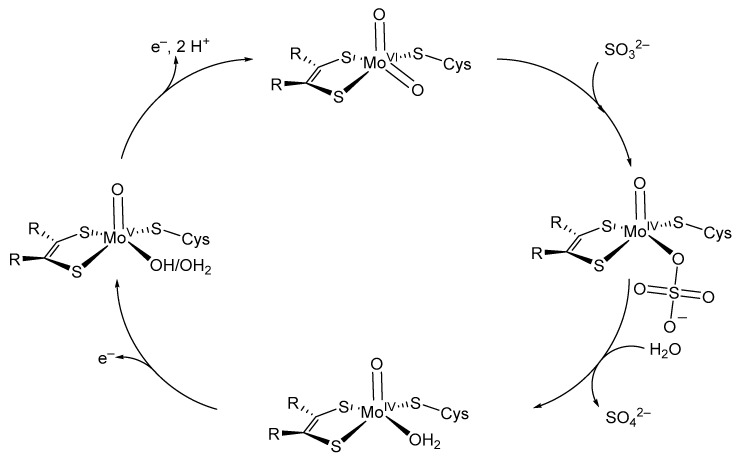
Catalytic cycle of sulfite oxidase with its two half reactions: the OAT (**right**) and the PCET (**left**).

The enzymatic oxygen atom transfer reaction (OAT) comprises two half cycles of reactivity: (i) the oxygen transfer from a substrate to the central metal ion accompanied by the oxidation of the latter; (ii) the metal’s two electron reduction typically coupled to proton transfer (PCET), or vice versa. Figure 3 shows, for instance, substrate oxidation by OAT and molybdenum oxidation through PCET. In enzymes either the reduction or the oxidation process utilizes water as the oxygen atom’s sink or source, respectively (Equation (1)) [11].

(1) $$R+H_{2}O⇄{\mathrm{RO}+2H}^{+}+2e^{-}$$

The oxygen atom transfer occurs at the metal center whereas the electron transfer chains include electron carriers, such as flavin, heme, or Fe-S-clusters, to restore the oxidation state of the metal center, which results in distinct reaction pathways [10,12].

In order to mimic the function of an enzyme, one is not compelled to precisely rebuild the active site structure. This means that researchers can establish new functional analogues by freely exploiting the huge variety of available chemical building blocks. The use of the dithiolene moiety is, hence, not a necessity, and bidentate [13,14,15,16,17,18], tridentate [19,20,21], or tetradentate [22,23,24] ligands with a mixture of oxygen, nitrogen, and sulfur coordination sites were introduced to replace the natural ligand system in functional analogues. Alternatively, the metal ion may also be varied. The molybdenum center can, for instance, be substituted by rhenium, due to the diagonal relationship between these two metals leading to an additional class of efficient OAT reagents [25,26,27,28,29]. Commonly, the examination of OAT reactivity includes natural and non-natural oxygen acceptors (organic phosphines, sulfite, selenite, arsenite) and oxygen donors (N-oxides, S-oxides), which still represent well-established, state-of-the-art approaches towards modelling either individual half reactions or entire OAT-only catalytic cycles when they are combined [21,22,23,27,30,31,32,33,34]. This means that a reduced substrate and an oxidized substrate are mixed with the functional model complex, which is taking the oxygen atom from one substrate and delivering it to the other without passing through any PCET.

### 2.1. Dithiolene Complexes

Considering the biological template of molybdopterin, bioinorganic chemists normally utilize simplified dithiolene systems to mimic natural reactivity. Respective synthetic analogues can be separated into two major groups depending on the number of dithiolene ligands present: mono(dithiolene) and bis(dithiolene) complexes. Tris(dithiolene) complexes can be easily synthesized, but are generally rather unreactive and therefore not commonly of interest. Mono(dithiolene) complexes are only rarely found as functional analogues in the existing literature due to their difficult syntheses and limited stabilities [8,35,36]. In contrast, bis(dithiolene) complexes represent a class of model compounds which are relatively reliably synthesized and concomitantly suitable synthetic analogues for OAT reactivity. Many strategies for ligand and subsequent complex synthesis have been published in the past [2,8,10]. These complexes are therefore versatile and well explored for their catalytic performance in the transfer of oxygen atoms.

A classical benchmark reaction for the determination of the OAT activity of a certain complex was developed by Holm and others [11,37]. The natural ping-pong mechanism involving water, substrate, and metal ion center is simulated utilizing S-oxides or N-oxides as oxygen donors and organic phosphines as oxygen acceptors. The simultaneous application of both substrates links two OAT reactions for which the appropriate kinetic rate constants can easily be determined.

**Figure 4:**
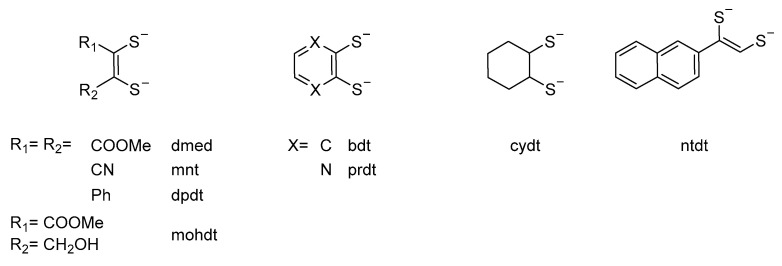
Selected dithiolene ligands used for the catalytic and the non-catalytic examination of oxygen atom transfer (OAT) reactivity.

During the last decade our group reported three of these bis(dithiolene) molybdenum complexes with comparably conveniently handled ligands 2-napthyl-1,4-dithiolate (ntdt), pyrazine-2,3-dithiolate (prdt), and 1-methoxy-1-oxo-4-hydroxy-but-2-ene-2,3-dithiolate (mohdt) (Figure 4). For the prdt ligand, a rhenium complex could also be synthesized and compared to its analogous molybdenum complex, exploiting the element’s diagonal relationship [27]. However, all these structural and functional analogues exhibited very slow oxygen transfer kinetics compared to previously tested complexes when using the ‘gold-standard’ DMSO/PR_3_ benchmark system developed by Holm. The observed reaction times for all these new complexes were in a range of several days and some did not even reach completion [27,31,32]. This slow rate can be attributed partly to the poor transfer reactivity of DMSO. For particularly potent catalysts, a deceleration of the reaction can be helpful in order to determine the kinetics, while for poor catalysts using better (quicker) substrates appears more reasonable. Replacing DMSO by the stronger oxygen donor TMAO indeed resulted in accelerated, though still not very impressive, conversions [32]. It was, hence, confirmed that bis-dithiolene complexes can exhibit very different and even surprisingly slow catalytic reaction rates in OAT transformations based on the ligands employed. Substituents of the dithiolene ligand systems induce electronic effects and thereby modulate the complex’ stability and reactivity, which counter balance each other and both affect the catalytic efficiency [38]. It was generalized before that electron-withdrawing groups pull electron density from the metal towards the ligand by weakening the Mo-dithiolene bond and consequently increasing the Lewis acidity, followed by the stabilization of the Mo=O bond, whereas electron-donating groups push electron density toward the metal center concomitant to a decrease in the Lewis acidity at the molybdenum and the mitigation of the Mo=O bond [39].

The mohdt ligand constitutes an interesting example with simultaneous electron-withdrawing and electron-donating functional groups at each end of the organic backbone, resulting in a push-pull effect that was sought to balance the charge transfer between metal and ligand [32]. However, this functional analogue did not perform as predicted, because complex dimerization became a significant problem that is associated with a temporary inhibition of the catalyst. This non-natural reactivity has troubled researchers since the early phase of model chemistry for molybdenum-mediated OAT reactions, because the concomitant presence of fully oxidized and fully reduced species often leads to the reversible formation of the Mo^V^ dimer (represented in Equation (2)) [11].

(2) $${\mathrm{Mo}}^{\mathrm{IV}}{\mathrm{OL}}_{n}{+\mathrm{Mo}}^{\mathrm{VI}}O_{2}L_{n}⇄{\mathrm{Mo}}_{2}^{V}O_{3}L_{2n}$$

This dimerization is predominantly observed for dithiolene ligands with an aliphatic (e.g., electron-withdrawing) backbone. It represents a resting state within the ping-pong cycle. Depending on the extent of the formation of the dimer, it can dramatically decrease the concentration of the actually active species. The observed very slow substrate conversion by the mohdt bearing functional analogue most likely goes back to such dimerization [11,32,37]. This emphasizes how tricky it is choosing the best set of substituents in order to design a structural dithiolene-bearing model of the oxidoreductases which also shows at least moderately good functional activity.

While the mohdt ligand provides an asymmetric electron density distribution within one ligand, Sarkar and others employed a different strategy. They synthesized mixed bis(dithiolene) molybdenum complexes. Each complex bears one maleonitrile-1,2-dithiolate-ligand (mnt) plus one additional dithiolene, either toluene-3,4-dithiolate (tdt), benzene-1,2-dithiolate (bdt) or 1,2-dicarboxymethoxyehtylene-1,2-dithiolate (dmed) [30]. The observed electron density exhibited an asymmetry over the entire complex in contrast to the mohdt-derived complex in which the effect is more localized. Notably, the combination of mnt with bdt resulted in a higher reaction rate than for the mnt/dmed system, regarding the oxygen atom transfer from TMAO to the complex, even though aromatic dithiolenes are not considered as giving particularly active catalysts. Complex composition was designed by the authors in order to address the distinct spatial orientation of the two MPT ligands in the active site of the DMSOR family. These different MPTs were identified in the crystal structure of DMSOR (there named P and Q pterin), and were suggested to lead to an asymmetric electronic modulation of the metal center which promotes reactivity [40,41,42,43].

Instead of applying Holm’s double OAT benchmark reactions and catalytic cycles as in the examples described above, important mechanistic and kinetic insight for functional models and, by extension, the natural active sites, can also be derived from the examination of ‘only’ their half reactions. In 2014, a mostly computational study (DFT supported by EXAFS) addressed the electronic differences in the active site reactivities of DMSO *reductase* and sulfite *oxidase* enzymes employing model compounds [44]. It was possible to theoretically differentiate comprehensive mechanistic details of the oxygen atom transfer from metal to substrate and from substrate to metal. Using DMSO as substrate and a monooxido molybdenum(IV) complex, an S=O bond elongation was found to lower the energy of the S-O antibonding orbital. This closes the gap between the molybdenum centered HOMO and the substrate centered empty orbital that is about to receive the two electrons. The actual OAT is, hence, initiated by the O-Mo contact, and the weakening of the O-S bond occurs prior to the transfer of the two electrons from metal to substrate. In case of the reverse OAT from metal to substrate, trimethylphosphite and a dioxido molybdenum(VI) center was investigated. Here the energy barrier is already smaller in comparison between the Mo=O centered LUMO (antibonding) and the lone pair of the oxygen acceptor phosphite, enabling swift orbital mixing and concomitant electron transfer. In case of the transfer from metal to substrate, the electron flow, therefore, precedes the oxygen atom transfer, i.e., the exact opposite compared to metal center oxidation.

Considering these notable mechanistic differences, it appears indeed reasonable to evaluate the two types of OAT, with regard to the direction of the transfer, separately. In the following, the focus is on OAT-capable complexes which model the half cycle reactions transferring the oxygen atom from metal to substrate, i.e., the conversion of M^VI^ to M^IV^. Sugimoto and others, remarkably, further extended their respective investigations beyond OAT, also looking at oxygen’s heavier congeners sulfur and selenium. The transfers of oxygen, sulfur, and selenium atoms to organophosphines were compared considering tungsten as well as molybdenum complexes as oxidants [33,45,46,47,48,49,50]. The investigations focused on mechanistic and kinetic details of these reactions. For the sake of comparability, the employed dithiolene ligand was in all cases the methylester derivative dmed (see Figure 4). It must be noted here that the preparation of these S and Se bound complexes is far from trivial and they are also very difficult to handle due to their lability. For the sulfido and selenido complexes, the desoxo [M^IV^(OR)L_2_]^−^ (OR = alcoholato ligand) species were employed as starting materials which were first reacted stoichiometrically with suitable N-oxides yielding the octahedral [M^VI^O(OR)L_2_]^−^ type complexes. In a second transformation, the alcoholato functionality was replaced by S or Se using EH^−^ as reactants (E=S, Se) [46,50].

**Figure 5:**
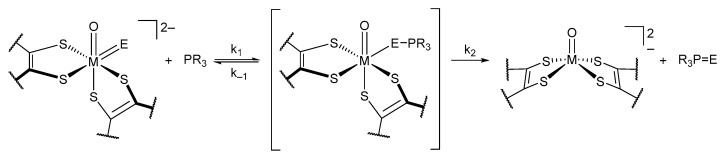
Mechanism of E atom transfer reactions (E = chalcogenide atom) from metal centers to organophosphines (PR_3_). The formal oxidation state of the metal changes from +VI to +IV; phosphorous is concomitantly oxidized. k_1_, k_−1_, and k_2_ represent the respective reaction rates. The figure was adapted from the original literature [38,46].

The mechanism and kinetics of the chalcogenide transfer to organophosphine are best described with a two-step reaction scheme (Figure 5); the overall observed pseudo first-order rate constants showed saturation curves with respect to organophosphine concentration. This implies that the dissociation of the product (R P=E) from the active site is the rate-determining step [46]. With regard to that particular observation, Fischer and Fischer in 2016 provided another perspective (derived from the already published data) and pointed out that the underlying fundamental reaction is a simple consecutive reaction with only the first step being actually dependent on the substrate concentration [38]. An increase in the substrate concentration accelerates the reaction rate of the first step, which eventually overruns the second step (substrate independent). The latter scenario would then lead to the observed “saturation type” kinetics. The transfer of oxygen (O^0^) to the substrate in the Sugimoto experiments occurred with very slow reaction rates which were explained as being the result of the strong electron-withdrawing effect of the dmed ligand system stabilizing the higher oxidation state. Comparative DFT calculations revealed the participation of the transferred E in the LUMO of the [M^VI^O(E)(dmed)_2_]^2−^ (M = Mo, W; E = O, S, Se) species and a significant respective difference for oxygen in contrast to sulfur and selenium. The contribution to the LUMO by the oxido ligand was only 9.74% for M = Mo and 1.41% for M = W, whereas sulfur (Mo: 34.42%; W: 34.45%) and selenium (Mo: 35.33%) held a much more substantial share of the LUMO [46,50]. As a result, the transfer rates were clearly enhanced in the cases of selenium and sulfur [46,50]. Additionally, the influence of the metal center with regard to sulfur atom transfer was investigated. While the molybdenum complex exhibited saturation type kinetics, the corresponding tungsten complex showed only a first-order dependency on the PR_3_ concentration. This defines the association between phosphorous and sulfur as rate-determining for the latter. The apparent second-order rate constant for the molybdenum complex (only the initial linear part of the saturation curve was considered) was nine times higher in comparison to the tungsten complex rate constant (k_2_: 15.4 M^−1^ s^−1^ vs. 1.7 M^−1^ s^−1^), which is in accordance with previous observations for this type of half cycle reaction, i.e., the oxygen atom transfer from complex to substrate and metal ion reduction: k_Mo_ > k_W_ [8,46].

**Figure 6:**
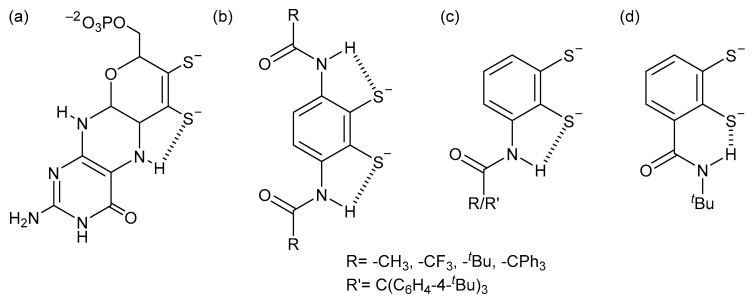
Comparison of the hydrogen bonding interactions in the molybdopterin moiety (**a**), with the different substituted aromatic ligand systems 1,2-S_2_-3-R-CONHC_6_H_3_ (2N-bdt, (**b**)), 1,2-S_2_-3-R-CONHC_6_H_3_ (N-bdt, (**c**)) and 1,2-S_2_-3-*^t^*Bu-NHCOC_6_H_3_ (O-bdt, (**d**)) introduced by the groups of Ueyama and of Onitsuka [51,52,60].

In the other type of half cycle reaction, a substrate donates its oxo function as an oxygen atom to the metal ion center accompanied by the oxidation of M^IV^ to M^VI^. Respective OAT donor reagents/substrates are often derived from trimethylamine-N-oxide (TMAO) since this is actually also a natural substrate for TMAO reductase, or dimethylsulfoxide (DMSO), as in the DMSO reductase enzyme [30,33,51,52]. In 2006, Ueyama and others designed a novel aromatic dithiolene ligand 1,2-S_2_-3-R-CONHC_6_H_3_ (2N-bdt; R=CH_3_, *^t^*Bu, CF_3_, CPh_3_, C(C_6_H_4_-4-*^t^*Bu)_3_) (Figure 6b), bearing two amide moieties adjacent to the dithiolene in order to enable intramolecular hydrogen bonding in their complexes since H bonds play important roles for the enzymes’ active sites and reactivities [53]. These interactions in the model compounds modulated the electronic bonding preferences, resulting in a positive shift of the redox potential. This shift goes back to the H bond supported stabilization of the dithiolene to metal ion coordination reflected in decreased M-S(dithiolene) bond lengths [54,55,56,57]. An observed asymmetric hydrogen bonding in the backbone of MPT was the inspiration for exploiting specifically the intramolecular H bonds in these model compounds (Figure 6a) [42]. As pointed out in the literature, the MPT ligand represents a redox relay in which the tetrahydropyrazine moiety can be oxidized to the dihydro or fully oxidized pyrazine ring system [2]. Hydrogen bonding interactions necessarily change, when the oxidation/protonation states of the nitrogen atoms are altered, which is particularly relevant for those N that lie adjacent to the dithiolene donor site. In their experiments, Ueyama et al. found an accelerated addition of the oxygen of TMAO to the [Mo^IV^O(2N-bdt)_2_]^2−^ complex in contrast to the non-substituted related benzene-1,2-dithiolate-derived complexes introduced by Garner and Clegg much earlier [53,58]. Following up on these findings, Onitsuka and others later changed the properties of the employed 2N-bdt ligands; they removed one amide moiety to obtain the asymmetric 1,2-S_2_-3-R-CONHC_6_H_3_ (N-bdt; R = CH_3_, *^t^*Bu, CF_3_, CPh_3_, C(C_6_H_4_-4-*^t^*Bu)_3_) (Figure 6c) and 1,2-S_2_-3-*^t^*Bu-NHCOC_6_H_3_ (CO-bdt) (Figure 6d) dithiolenes and investigated the *trans* influence which had been noted for the tungsten 2N-bdt analogue [52,59,60]. The results showcase similarly enhanced reactivity for all complexes utilizing amide-bearing dithiolene ligands in comparison to complexes with bdt. Respective kinetic features were investigated using UV/vis spectroscopy with a complex to substrate ratio of 1:2. N.B., the reaction was treated as being pseudo-first order to derive rate constants, which is not kinetically correct for the applied (relative) concentrations; this should be taken into consideration when discussing or comparing future studies to these kinetic results. The observed differences and similarities among these systems are still very interesting and relevant, though. Model complexes with these asymmetric dithiolene ligands that have inherent hydrogen bonding properties may enable a ‘guided’ association of substrate and metal center plus a follow-up change in orientation, which is thought to improve the binding of a suitable oxygen acceptor molecule, i.e., acting as a miniature bioinspired hydrophobic enzyme-like pocket [51].

Within the DMSOR family, TMAO reductase is coordinated to the peptide by a serine residue, whereas for nitrate reductase (NR), aspartate or cysteine were detected in the first coordination sphere of molybdenum [61,62]. The latter converts a chemically less reactive substrate, implying that the NR enzymes possess increased reactivity [10,33,63]. Using alcoholato and thiolato desoxomolybdenum(IV) complexes as models, Sugimoto et al. investigated the potential influence of these peptide-based chalcogen donor atoms in the respective enzymes [33]. Donor atoms as well as steric influence were studied with the oxygen donation from different N-oxide substrates. Following previously utilized strategies for systems with 1,2-dimethylethylene-1,2-dithiolate or 1,2-diphenylethylene-1,2-dithiolate ligands [63,64,65,66,67], an additional aliphatic ligand system was introduced, cyclohexene-1,2-dithiolate (cydt, Figure 4), with relatively strong electron-donating properties. A molybdenum complex bearing two cydt and one phenolato ligand exhibited notably faster OAT transformations than all other complexes from this comparative study. The acceleration was attributed (a) to the strong electron-withdrawing properties of the ligand and the resulting increased Lewis acidity at molybdenum and (b) to the reduced steric strain (vs. 2,6-diisopropylphenolate) of the alcoholato ligand. The reaction accelerated further when the chalcogen donor was changed from oxygen (alcoholate) to sulfur (thiolate). This observation was explained by the authors with reference to the larger contribution of the sulfur donor atom p-orbital to the coordinative bond as reflected in the distinct bond angles, 107.2° for the Mo-S-C and 160.5° for the Mo-O-C bond, respectively, which also facilitates the access of the substrate to the vacant metal coordination site in case of the thiolate ligand; i.e., a mix of interdependent electronic and steric effects is responsible [33].

**Figure 7:**
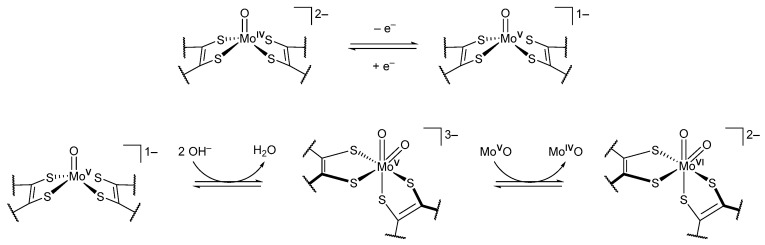
Proposed mechanism for the artificial PCET reaction with Mo^IV^OL_2_ (L = bdt, dmed). The reaction cascade starts with an electrochemical activation (**top**) followed by a consumption of two hydroxide ions to produce a Mo^V^O_2_L_2_ complex which can disproportionate, resulting in a Mo^VI^O_2_L_2_ species (**bottom**). The figure was adapted from [48].

In the examples discussed above, direct OAT reactions from or to a suitable substrate have been in the center of attention. However, this refers only to one half reaction of the natural mechanism. The other half of the natural enzymatic catalytic cycle, its regenerative part, utilizes water through a double PCET reaction as natural source or sink of oxygen [11]. Sugimoto and others investigated an artificial PCET model system in which the [Mo^IV^OL_2_]^2−^ (L = dmed, bdt) complex was electrochemically oxidized together with spectroscopic reaction monitoring of the Mo^V^ species in presence of hydroxide ions in acetonitrile solvent [48]. The product of the reaction cascade was identified as the Mo^VI^O_2_L_2_ analogue. The reaction pathway comprises the consumption of two hydroxide ions followed by a disproportionation probably going through the µ-oxo-dimer intermediate (Figure 7). In contrast to related work of Xiao et al., who employed trispyrazolylborate (Tp^−^) ligands instead of dithiolenes [68], the intermediate species Mo^V^O(OH) and Mo^V^O_2_ could not be isolated. Changing the ligand system obviously interfered with the overall kinetics of the reaction cascade, which likely originates from the much better steric protection of the Tp^−^ ligand and possibly a variation in the Lewis acidity of the metal center, due to the different donor atoms and local charges. The kinetic and electrochemical data of Sugimoto et al. implied a stronger electron-withdrawing effect for the dmed ligands compared to the bdt ligands, which facilitated the nucleophilic attack of the hydroxide ions [48]. Since modelling the conditions in which PCET occurs in nature requires (at least moderately) aqueous solvents and specific pH values, these modelling experiments are exceptionally challenging and consequently very rarely found in the literature.

Extremophilic organisms live in harsh environments under extreme conditions. Considering their enzymes, reactivities of functional analogues should ideally be assessed with the application of typical environmental parameters (temperature, pH, ion concentrations, etc.). Kim and others, accordingly, investigated a model for tungsten-dependent formate dehydrogenase (W-FDH; from thermophiles) under, respectively, harsh, i.e., high temperature, conditions [69]. The well-known 1,2-dimethylethylene-1,2-dithiolate (mdt) ligand was used to synthesize the corresponding [WO(mdt)_2_]^2−^ complex. When dissolved in acetonitrile and exposed to 90 °C in a CO_2_ atmosphere (and apparently in presence of a proton source), it formed an unexpected dinuclear (Et_4_N)_2_[W_2_^V^O_2_(µ-S)(µ-mdt)(mdt)_2_] species after four days. The origin of the protons could not be completely validated though acetonitrile was strongly suspected. Other products of the reaction, formate (CO_2_ substrate derived as expected) and dimethylthiirene (apparently from dithiolene) were detected by GC-MS [69]. The normally well-behaved [WO(mdt)_2_]^2−^ engaged in substantially different reactivity at the applied high temperature, which emphasizes the difficulties encountered when modelling extremophilic enzymes and reactions.

Besides mimicking harsh environmental conditions, replacing oxygen by sulfur in the sink/source of the transferred chalcogen atom represents another example of a research issue that is comparably difficult to address. These efforts fall into the context of a rarely examined enzyme: polysulfide reductase. This enzyme is found in anaerobic prokaryotes (e.g., *Wolinella succinogenes*) and fuels ATP synthesis. Hence, it facilitates so-called polysulfide respiration, in which an electron donor (formate or H_2_ from FDH or hydrogenase, respectively) enables the enzymatic reduction of polysulfide to sulfide (Equation (3)) [70,71].

(3) $$S_{n}^{2-}+{2H}^{+}+{2e}^{-}⇄S_{n-1}^{2-}+H_{2}S$$

Very few respective and at least fractionally functional analogues are reported in the literature. The groups of Sarkar and later Sugimoto introduced molybdenum and tungsten complexes which were found to be reactive toward polysulfide and sulfur, respectively [72,73,74]. However, the coordination of sulfido ligands is accompanied by increasing instability and reactivity as reflected in the faster sulfur atom transfer (SAT) compared to OAT (*vide supra*). Sugimoto and others established a new tungsten containing synthetic analogue [W^IV^SL_2_]^2−^ with 1,2-diphenylethene-1,2-dithiolate (dpdt, Figure 4) as a ligand system which was able to activate three equivalents of sulfur to yield a [W^IV^(S_4_)L_2_]^2−^ species. A polysulfide-like product η^1^-S_3_^2−^ was discussed as an interim ligand possibly oxidizing the tungsten from +IV to +VI. This would result in an intermediate which can be rearranged by reducing the metal back to +IV and fusing the two sulfur coordination sites S^2−^ and S_3_^2−^ to give S_4_^2−^.

Complementing the traditional synthetic and catalytic chemical approaches, electrochemical and photochemical applications allow certain bioinorganic questions about biomimetic catalysis, e.g., when modelling the biological electron transfer chains to regenerate the active sites, to be addressed. This would be enormously difficult with solely chemical methods. Due to the rising interest in reducing the greenhouse gas CO_2_, molybdenum and tungsten-containing formate dehydrogenase (FDH) as well as the Cu/Mo bearing CO dehydrogenase 2 (CODH2) became particularly important study targets. Enhancing enzyme reactivity for biotechnological application and synthesizing efficient functional analogues of the active sites are two major lines of research in this context [75]. Electrochemical and photochemical methods have been widely used for modulating the electronic properties of metal centers in chemical compounds to instigate redox catalysis. Fontecave and others combined different dithiolene ligands with molybdenum and nickel ions as active analogues for FDH and designed a unique bimetallic compound with molybdenum and copper ions in a single model complex for CODH2 [76,77,78,79,80].

**Figure 8:**
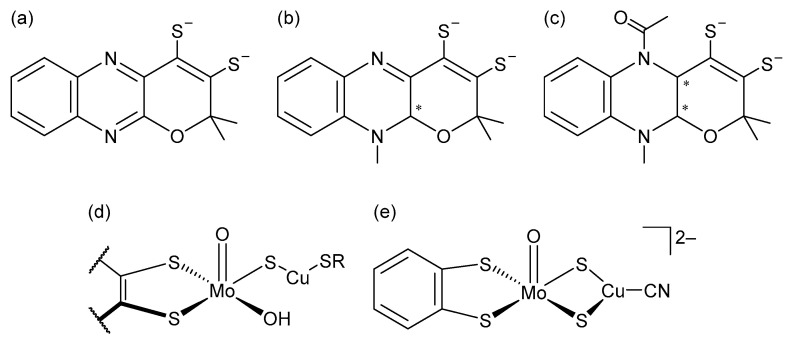
(**a**–**c**) Employed ligand systems for FDH functional analogues based on a fused quinoxaline-pyrane ring system with oxidized (qpdt, (**a**)), semi-reduced (H-qpdt, (**b**)) and fully-reduced (2H-qpdt, (**c**)) pyrazine/piperazine ring, respectively (* represents chirality), (**d**,**e**) comparison of the active site of MoCu CO dehydrogenase 2 (MPT only shown as dithiolene) (**d**) with the synthetic analogue (Et_4_N)_2_[(bdt)Mo^VI^O(µ-S)_2_Cu^I^CN] (**e**) introduced by Fontecave et al. [76,78,84].

The ligand systems applied here are relatively more similar to MPT than the examples discussed above. They comprise tricyclic systems in three conformations, i. oxidized (qpdt, Figure 8a), ii. semi-reduced (H-qpdt, Figure 8b) and iii. fully reduced (2H-qpdt, Figure 8c), in which a pyrazine/piperazine ring is fused to a benzene ring on one side and to a pyrane ring (bearing the dithiolene coordination donor site) on the other side. Similarly advanced ligand systems were originally introduced jointly by the groups of Garner and Joule in 1998. However, they never succeeded in unambiguously coordinating molybdenum centers with their ligand systems, whereas the analogous cobalt complex [Co^III^Cp(dt)] (Cp = cyclopentadienyl; dt = dithiolene) turned out to be even air-stable [81,82,83]. Based on this seminal work, Fontecave et al. synthesized related CoCp-dithiolene complexes with the three ligands described above. They were, most notably, also able to prepare the three corresponding molybdenum monooxido bis(dithiolene) complexes [76,77,78]. An unusual observation was made when analyzing the molecular structure of the molybdenum 2H-qpdt analogue: molybdenum was found to be in the formal oxidation state +V, not +IV as expected [76]. The three molybdenum complexes were examined for their ability to reduce carbon dioxide in a photocatalytic setup with [Ru(bpy)_3_]^2+^ as photosensitizer (10 equiv.) and 1,3-dimethyl-2-phenyl-2,3-dihydro-1*H*-benzoimidazole (BIH) as sacrificial electron donor in acetonitrile/triethanolamine (5:1). All complexes catalyzed the reduction of CO_2_ to formate and CO with competitive proton reduction. The presence of the more oxidized qpdt and H-qpdt ligands was found to lower the selectivity, as their complexes produced more hydrogen byproduct (substrate selectivity CO_2_/H^+^: 0.23 and 0.86, respectively). The molybdenum complex bearing the reduced 2H-qpdt (which would be expected to increase the complex’s reducing power) showed the highest selectivity for CO_2_ (CO_2/_H^+^ = 1.38). The same ligands were used for the synthesis of the respective nickel bis(dithiolene) complexes, which exhibited similar reactivity. For this study, electrocatalysis was chosen as the method and the 2H-qpdt complex showed again the best selectivity towards transforming CO_2_ to formate and CO with very low competitive hydrogen evolution [79,80]. Complexes with the same ligand but different metal center (molybdenum vs. nickel) exhibited notable differences. The nickel complex with 2H-qpdt was more selective toward CO_2_ reduction (faradaic yield after 20 h: carbon species = 72%; TON = 90), whereas the molybdenum complex was found to be more active with a higher turnover number (TON) (after 15 h: carbon species = 60%; TON = 123) [79]. However, since the complexes with the distinct metals are not structurally analogous, and because different activation methods were used (photo vs. electro), conclusions derived from that comparison should be taken with caution.

The natural molybdenum containing CODH2 enzyme was found to not convert CO_2_ as in the FDH enzymes but to irreversibly oxidize CO, which is not fully understood as of yet [85]. Modelling the reactivity of this very unusual active site (Figure 8d) comprises a challenging task. Initiated by Tatsumi and others in 2005, only very few functional analogues can be found in the literature; some bear dithiolene ligands [84,86,87] while others are dithiolene-free [88,89,90]. The Mo/Cu complex of Fontecave (Figure 8e) [Mo^VI^O(bdt) (µ-S)_2_Cu^I^CN]^2−^ represents the first example of a synthetic model complex for the active site of CODH2 which reduces CO_2_ via electroreduction and thereby reverses the natural reactivity. In the presence of CO_2_ and a proton source (trifluorethanol), the catalyst selectively generated formate as major product, with CO and H_2_ as side products in good faradaic yields (96% after 2 h; TON = 4; selctivity 69% formate vs. 8% CO vs. 19% H_2_) [84].

**Figure 9:**
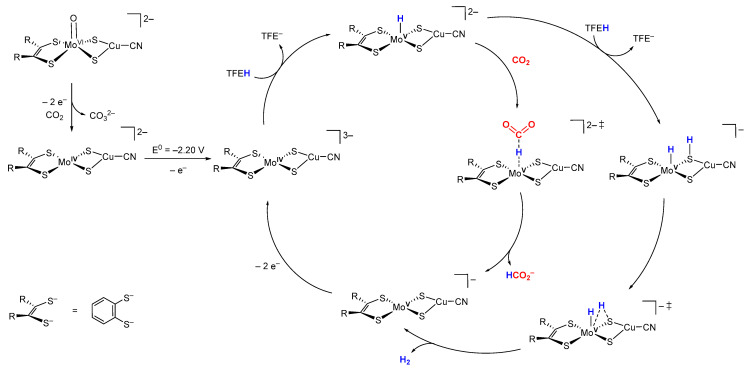
Proposed catalytic cycle for CO_2_ activation yielding formate and H_2_ by the novel (Et_4_N)_2_[(bdt)Mo^VI^O(µ-S)_2_Cu^I^CN] complex, a functional analogue of the Mo/Cu CO dehydrogenase 2. The standard potential in the second step is given vs. Fc^+^/Fc and constitutes a one-electron reduction into the ligand-based molecular orbital with almost exclusively S_3p_-character; TFE = Trifluorethanol. Adapted from the original report [84].

Digging deeper, the authors applied infrared (IR) spectroelectrochemical (SEC) methods supported by DFT computations for insights into the catalytic cycle. The results indicate the presence of carbonate, implying a bifunctional nature of CO_2_ in the catalytic transformations. It not only served as general substrate, but also facilitated formation of the active catalyst. The activation process comprises formation of carbonate from CO_2_ and the oxido ligand of the molybdenum complex (a classic example of OAT) plus electrochemical reduction. Subsequently, an intermediate square planar molybdenum(IV) species with a triplet ground state (confirmed by DFT computations) converts into the active catalyst via electrochemical reduction. Notably, an in situ Mo-H species was detected and interpreted as the cross-section for proton reduction and formate generation (Figure 9). Further investigations confirmed a critically important role of the Cu-site; in its absence, the corresponding molybdenum bis(dithiolene) complex [MoO(bdt)_2_]^2−^ was inactive [84].

### 2.2. Non-Dithiolene Complexes

The model chemistry for molybdenum and tungsten containing oxidoreductases is dominated by dithiolene ligand systems for their structural (and electronic) similarity to the natural MPT. However, their *non-innocence* character and proneness to be oxidized induce a sensitivity in the target complexes and, even more so, in reaction intermediates. As a consequence, this will lead, for instance, in the redox-comproportionation, to catalytically inactive dimers (see Equation (2) above) [10]. Replacing dithiolene ligands by non-dithiolene ligands when addressing research issues that are unrelated to the specific role of MPT comprises therefore a reasonable and certainly justified strategy.

One active site component of particular interest is the so-called ‘spectator’ oxido ligand, for instance, which is not uniformly present in the various cofactors of the different oxidoreductase enzymes [22]. The label ‘spectator’ is used to characterize this oxygen as not being transferrable; i.e., its presence only modulates the active site’s chemical and electronic structure. Functional analogues bearing such spectator oxido function in the absence of any dithiolene ligands are widely used and come with a huge variation in structural properties and ligand types (e.g., bi-, tri- or tetradentate). Reactions, such as oxygenations and epoxidations of non-natural substrates, widen the reactivity scopes of these complexes substantially. However, looking at non-natural transformations of non-natural substrates would go well beyond the aim of this review, which is to highlight bioinspired functional models for oxidoreductases. Readers interested in these systems/reactivities may refer to respective articles available in the literature [91,92,93,94,95,96,97].

Some of the most successful and most reliable ligands used in chemistry belong to the tridentate scorpionate ligand class: hydrotrispyrazol-1-yl-borate (Tp), hydrotris(3,5-dimethylpyrazol-1-yl)borate (Tp*) and hydrotris(3-isopropylpyrazol-1-yl)borate (Tp*^i^*^Pr^). These ligands together with their corresponding molybdenum complexes were predominantly investigated by Trofimenko for a considerable time span between the years 1967 and 1989 [98,99,100,101,102]; these systems continue to play an important role in more modern OAT research. The history and utilization of scorpionate complexes as chemical analogues of molybdenum enzymes are expertly illustrated in a review article by Young, which is highly recommended [103]. Reported by Enemark, Young and others in the 1990s, a Tp* molybdenum complex was the first functional analogue that was able to catalyze sufficiently well a reaction between PPh_3_ and water as oxygen source [68,104]. The literature output for functional Mo and W model complexes with the Tp ligand and derivatives thereof has notably decreased throughout the last decade, though, implying that these ligands are no longer perceived as indispensable for the ongoing research activities in this context [19,105].

**Figure 10:**
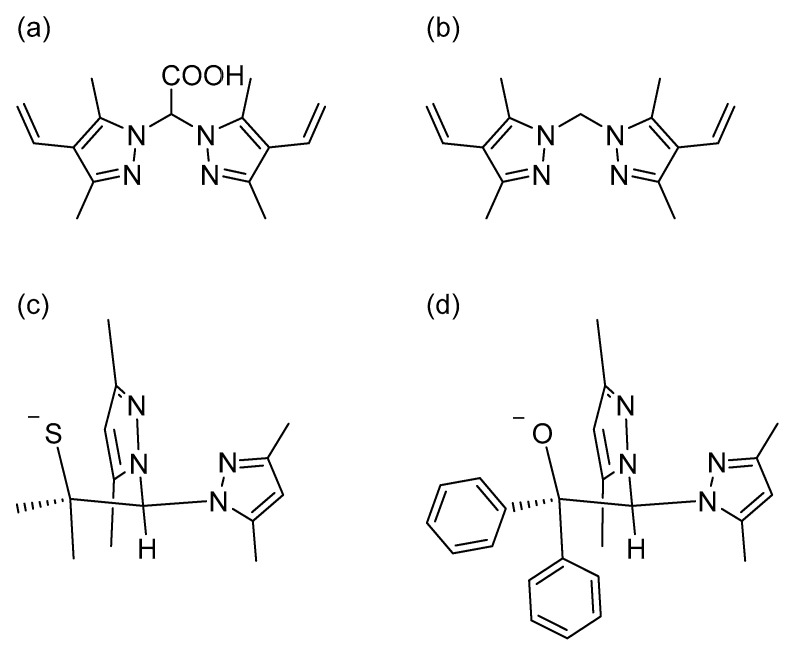
Scorpionate ligands bis(3,5-dimethyl-4-vinylpyrazol-1-yl)acetic acid (bpa, (**a**)) and bis(3,5-dimethyl-4-vinylpyrazol-1-yl)methane (bpm, (**b**)) used for homogenous and heterogenous OAT catalysis. The vinyl groups are connected to acrylate linkers resulting in a polymer [105]. Heteroscorpionate ligands based on Tp* with a thiolate (L3S, (**c**)) or an alcoholate (L10O, (**d**)) replacing one pyrazolyl moiety [19].

Still, the group of Carrano employed two heteroscorpionate ligands based on Tp* in which one pyrazolyl moiety was replaced by either a thiolate (L3S) or an alcoholate (L10O) substituent, respectively, to synthesize the corresponding [MoO_2_LCl] complexes (L = L3S, L10O) (Figure 10c,d) [19]. The transferrable oxido ligand of the molybdenum complex with the L10O ligand is located in either *trans* or *cis* positions to the spectator oxido ligand, whereas the thiolate analogue was only found as *cis* isomer. All three complexes/isomers were examined as stoichiometric OAT donor reagents with PPh_3_ as O^0^ acceptor. The *trans* isomer of the bulky L10O system showed no formation of the inactive µ-oxido dimer in addition to the transfer reaction product, which was attributed to steric bulk, and is an advantage over the L3S ligand with less steric strain. The L10O *cis* isomer was entirely inactive in the typically used solvents (acetonitrile, DMF) but active in pyridine albeit with rather slow reaction rates compared to the other two complexes. Additionally, *trans*-[MoO_2_(L10O)Cl] and *cis*-[MoO_2_(L3S)Cl] were catalytically active in DMF/d_6_-DMSO (1:1) with excess PPh_3_ [19].

The formation of µ-oxido dimers is a similarly challenging problem in the dithiolene as well as in the non-dithiolene model chemistry. When designing complexes with alternative ligands, these are, therefore, often chosen with regard to their presumed or apparent ability to impair such dimerization. One strategy involves the immobilization of the metal center on a polymeric ligand with multiple binding sites, essentially mimicking active site pocket characteristics [106,107]. Burzlaff and others fused a scorpionate ligand system to vinyl moieties (bis(3,5-dimethyl-4-vinylpyrazol-1-yl)methane = bpm, Figure 10b; bis(3,5-dimethyl-4-vinylpyrazol-1-yl)acetic acid = bpa, Figure 10a) to facilitate their polymerization with an acrylate linker (methyl methacrylate = MMA; ethylene glycol dimethacrylate = EGDMA) resulting in four different polymers [105]: i. bpm + MMA, ii. bpm + EGDMA, iii. bpa + MMA and iv. bpa + EGDMA. To synthesize the heterogeneous catalysts, the polymers were charged either with MoO_2_Cl_2_ or a molybdenum scorpionate complex, resulting in eight different molybdenum-bearing polymers. The catalytic activity of these polymeric species was then compared to the homogenous molybdenum scorpionate complexes [MoO_2_(bpm)Cl_2_] and [MoO_2_(bpa)Cl]. Notably, the homogenous catalysts showed much better OAT activity compared to the polymers and, among polymers, higher turnover numbers were observed when the MMA linker was used as co-monomer. Regardless of the absence (heterogenous catalysis) or probable presence of µ-oxo dimers (homogenous catalysis), however, the polymer approach had no apparent advantage [105].

**Figure 11:**
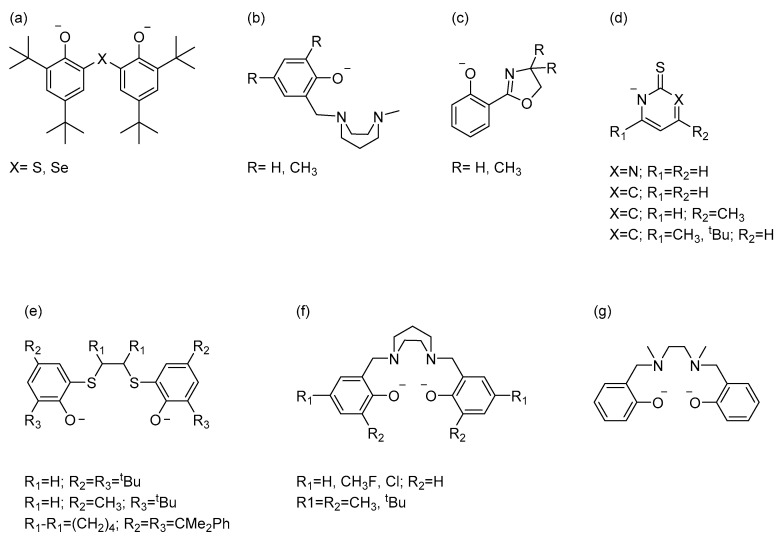
Selected non-dithiolene ligand systems used for OAT catalysis: (**a**) 2,2′-chalcogenobis(4,6-di-*tert*butylphenol) (Cbbp) [20], (**b**) 1,4-diazepane-based mono(phenolate) [21], (**c**) 2-(2′-hydroxyphenyl)-2-oxazoline (hoz) [25,109], (**d**) pyrimidine- (PymS) and pyridine-2-thiolate (PyS) [15,108], (**e**) 1,2-dithiaalkanediyl-2,2′-bisphenolate (OSSO) [23], (**f**) 1,4-diazepane-based bis(phenolate) [22], (**g**) ethylenediamine-based-bis(phenolate) [22].

Besides the historically important scorpionate complexes, several other catalytic systems were shown to facilitate full OAT cycles while employing a broad range of ligands (Figure 11) [15,20,21,22,23,108,109]. With the standard OAT benchmark catalysis introduced by Holm (*vide supra*), with S-oxides and PPh_3_ as substrates and with catalysts having specific modifications, the roles of metal centers (Mo vs. W) of donor atoms, and even of backbone substitution (second and outer coordination spheres) may be elucidated. For an evaluation of molybdenum vs. tungsten, 2,2′-chalcogenobis(4,6-di-*tert*butylphenol) (CbbP, Figure 11a) and 1,4-diazepane-based mono(phenolate) (Figure 11b) ligands were used for complexation. The tungsten functional analogue turned out to show better catalytic efficiency compared to the molybdenum complex [20,21]. This result is noteworthy, because normally the oxygen transfer reactivity of the oxido M^VI^ complexes is better for molybdenum than for tungsten (and vice versa for the oxidation of the M^IV^ species). Replacing the oxygen donor substrate by nitrate reversed the observed relative catalytic activity [20]. It was concluded that the tungsten analogue is almost unreactive regarding the oxygen transfer to the organophosphine (by which the metal is reduced). It shows, however, an improved overall OAT activity when a strong oxygen donor pushes the system forward (in the direction preferred by tungsten). The push then also supports the less reactive oxygen transport from W^VI^ toward the PR_3_ substrate. This is reflected in observations regarding the reactivity toward organophosphines made by Holm and Berg as well as Young, for which the latter declared the similar bond energies of W=O (>138 kcal/mol) and P=O (ca. 126–139 kcal/mol) to be at least partially responsible for the extremely poor reactivity [110,111,112,113].

With a focus on the ligand sphere rather than the central metal, Mösch-Zanetti and others investigated the impact of steric factors on OAT catalysis utilizing tetradentate 1,4-diazepane-like bis(phenolate) (Figure 11f) and salan-like bis(phenolate) ligand systems (Figure 11g) [22]. Notably, the complexes with the diazepane-like ligand [MoO_2_L] developed a rarely found *cis-β* conformation regarding the oxido moieties, which renders the two oxido ligands substantially distinct. The *cis-β* complexes exhibited an enhanced catalytic activity compared to those with salan-like ligands in a *cis-α* conformation. Furthermore, increasing steric hindrance by substitution in the *meta* positions (relative to alcoholato) resulted in catalytic inactivity, because the molybdenum coordination site for substrate is thereby sufficiently blocked. The electron-shift preference (push or pull) of the *para* substituent was further identified as having an impact on catalytic efficiency. Catalysis was improved with more electron-withdrawing groups in *para* position relative to the coordinating alcoholato substituent [22].

Work by Enemark back in 1985 later inspired the group of Okuda to employ the tetradentate 1,2-dithiaalkanediyl-2,2′-bis(phenolato) ligand (OSSO; Figure 11e) and an all-sulfur version thereof, 1,2-dithiaalkanediyl-2,2′-bist(hiophenolato) (SSSS) [23,114,115]. Some of the respective molybdenum complexes carry halides instead of the spectator oxido ligand resulting in desoxo Mo^IV^ and monooxo Mo^V^ species, respectively. The [Mo^VI^O(OSSO)Cl_2_] was unstable and found to rapidly convert to the [Mo^V^O(OSSO)Cl] complex. The latter represents the presumed intermediate during catalytic turnover which is deactivated in absence of the oxygen acceptor PPh_3_. In comparison to other model catalysts, the desoxo Mo^IV^ complex only performed at elevated temperature (60–80 °C, solvent dependent), though it was still more effective compared to the Mo^VI^O_2_ analogue. The dimerization process as proposed by Enemark was not observed for the [MoO_2_(SSSS)] system [114]. Surprisingly, the authors measured the highest catalytic activity for their molybdenum precursor [MoCl_4_(NCMe)_2_] at elevated and room temperature [23]. This is in accordance with OAT activity of other simple molybdenum complexes, such as [MoO_2_Cl_2_(dmf)_2_] or [MoO_2_(SCN)_4_]^2−^, which are commonly used as precursors for synthetic analogues, but also have a use as catalysts in industrial-scale deoxygenations [116].

Complementing these insights into the impact of the catalytically active species, a variation in substrates can similarly result in information about reactivity and mechanisms. In OAT catalysis, the oxygen acceptor substrate naturally influences the activity of the catalyst and the overall reaction rate. Most commonly, organophosphines are used considering the strong oxophilicity of phosphorous. Replacing PPh_3_ by PMe_3_ resulted in a decrease in reactivity for molybdenum complexes bearing pyrimidine- (PymS) and pyridine-2-thiolate (PyS) ligands (Figure 11d) introduced by Mösch-Zanetti et al. [15]. In contrast to the bulkier PPh_3_, the PMe_3_ molecule coordinates to the reduced molybdenum(IV) center, resulting in a reversible inactivation of the catalysts which could be countered with an excess of the oxygen donor substrate (S-oxides). Furthermore, dimerization was inhibited since PMe_3_ blocked the respective coordination site, as similarly reported by the Heinze group [17,18]. Complete inactivation was previously observed for some model complexes with salan-type ligands [22]. Regarding the PymS- and PyS-derived systems, this study implies a preferable use of PPh_3_ as oxygen acceptor, although it facilitated the formation of the µ-oxido dimer which was additionally synthesized. The dimerization process can be hindered through fine-tuning of the ligand system and enhancing catalysis using more potent S-oxides as oxygen donors [15]. The group of Hardré also described the dimerization process for an OAT catalyst bearing the PyS ligand which was generated in situ from an air-stable molybdenum pre-catalyst coordinated by the corresponding N-oxide (OPyS) [108].

Normally, the PR_3_/DMSO combination works sufficiently well as donor-acceptor substrate mix for monitoring and investigating the OAT reactivity and evaluating the activity of different catalysts. However, there are other opportunities, aside from PR_3_, to establish a metal-reducing pathway without using a direct oxygen acceptor. As an alternative, benzoin can be utilized. This substrate has the advantage of not only reducing the metal center but also concomitantly producing water, and mimicking the regeneration of the active site in the enzyme [117,118,119]. As downside, high temperatures had to be applied (80–100 °C) and the generated water can also be viewed as disadvantage for the known instability of the employed complexes against hydrolysis. The Avecilla group carried out some OAT catalyses using the DMSO/benzoin pair with several molybdenum complexes bearing tetradentate diamine-bis(phenolate) ligand systems reaching TOF values of 22–46 h^−1^. In all cases, dimerization was detected while it represented clearly a reversible resting state with no actual harm for the catalytic transformation (*vide supra*) [24,120].

N-oxides and S-oxides represent the natural substrates for the enzymes TMAOR and DMSOR and their wide use in these catalytic studies is well justified. It became necessary, however, to expand the range of employed substrates considering, in particular, the nitrate and perchlorate anions, which are metabolized by molybdenum-containing oxidoreductases, namely perchlorate reductase, chlorate reductase and nitrate reductase being members of the DMSOR family [121,122]. The conversion of the respective substrates contributes to oxygen-free respiration chains used by anaerobic bacteria yielding energy equivalents, such as ATP, needed to power metabolism. The pathway for perchlorate reduction plus the involved enzymes are provided in Scheme 1. The first two steps are catalyzed by molybdenum-dependent PcrAB, the target of respective molybdenum model chemistry. The utilization of nitrate (denitrification) can lead into different directions (Scheme 2) [123,124]. The typical denitrification results in the formation of molecular nitrogen from nitrate via nitrite, nitric oxide, and nitrous oxide. The dissimilatory nitrate reduction yields ammonium as product of nitrate in, potentially, a single step transformation [125]. Anaerobic ammonium oxidation (Anammox) also generates nitrogen, but forms hydrazine as an intermediate, resulting from a comproportionation of nitric oxide and ammonium [124].

Accumulation of nitrate (source: agriculture) and perchlorate (source: rocket fuels, military, pyrotechnique) in the groundwater represents a serious problem; health issues have been reported in connection with the uptake of contaminated drinking water [123,126,127,128,129]. As a result, the development of efficient catalysts targeting specifically the removal of nitrate and perchlorate as pollutants from water has become ever more important. However, functional analogues utilizing molybdenum with dithiolene or non-dithiolene ligands are notably scarce. In both substrate cases, the first effective homogenous catalyst was a rhenium species: [ReOCl(hoz)_2_] (hoz = 2-(2′-hydroxyphenyl)-2-oxazoline, Figure 11c) for perchlorate reduction [130] and a methyl rhenium oxide [Re(CH_3_)O_2_] for nitrate and perchlorate reduction [131]. Another example for an active homogenous perchlorate and nitrate reducing catalyst is an iron complex reported by Fout et al. [132,133].

For functionally modelling nitrate reductase, the previously reported molybdenum complexes with the CbbP ligand (Figure 11a) as well as the PymS and PyS systems showed catalytic activity towards nitrate reduction, albeit inefficiently, when PPh_3_ was used as oxygen acceptor [15,20]. The catalytic rate of the CbbP-bearing system reflects slow conversion when compared to the well-established PPh_3_/DMSO system (k_DMSO_ >> k_nitrate_) [20]. Catalysis with the PymS and PyS complexes stopped after 2–6 h with low conversion rates (12–22%) which was attributed to the concomitant formation of isolable nitrosyl species [15]. However, replacing molybdenum by rhenium and employing a modified hoz ligand (methylation of the oxazoline ring, compare to Figure 11c) leads to sufficient conversion of ^15^N labeled nitrate to nitrite after 5 h with DMS as oxygen acceptor [109]. After 48 h, the only detectable nitrogen species was nitrous oxide, which implies that this system is a potential artificial denitrification catalyst for the transformation of nitrate to N_2_O. Notably, the enzymatic conversion of nitrite to N_2_O is performed by a copper dependent oxidoreductase [124].

A molybdenum complex with the bidentate bis(4-*tert*-butylphenyl)-2-pyridylmethanethiolate (SN) ligand was investigated by Kim and others and shown to reduce nitrate, but only in the presence of non-catalytic amounts of scandium(III) ions. The Sc^3+^ ion acts as a Lewis acid to polarize the nitrate ion and consequently weakens the N-O bond, which then interacts with the molybdenum center [134]. As a side effect, the additive was supposed to reduce the nitrite produced in a molybdenum independent reaction to nitrous oxide.

Addressing perchlorate reduction, the Mösch-Zanetti group put a lot of effort into investigating homogenous catalysts with rhenium as metal center using the hoz ligand system (Figure 11c) initially developed by Abu-Omar et al. and further applied different tetradentate iminophenolate ligands together with molybdenum, which resulted in the first bioinspired homogenous molybdenum catalysts supported by PymS and PyS ligands (Figure 11d), respectively [25,26,130,135]. Additionally, reports in the literature exist of molybdenum-based heterogeneous catalysts on Pd/C platforms for aqueous (per)chlorate reduction; however, this falls beyond the scope of this review [136,137,138].

The rhenium catalyst with the bidentate hoz ligand showed a notably high conversion rate for the oxidation of the uncommon substrate diphenylsulfide (DPS) to diphenylsulfoxide (DPSO) and the reduction of perchlorate. It was assumed that perchlorate was fully reduced to the chloride due to the observed 87% conversion of the four equivalents of DPS. Surprisingly, the adapted coordination mode of the hoz ligands influenced the reaction rate of the complex catalyst; the N,N-*trans* isomer developed an increased turnover compared to the N,N-*cis* isomer (k_saturation_ [min^−1^]: 6.92 vs. 1.67) [25]. Adjacent nitrogen atom coordination was assumed to be responsible for the slow conversion by the rhenium complex with the tetradentate iminophenolate ligands. Adjusting the rigidity of the bis(imine) backbone changed the orientation of the O=Re-Cl axis, which exhibited *trans*-coordination with increased stiffness and vice versa for the *cis-*coordination. In consequence, the *cis*-isomer was the only sufficient catalyst. However, this functional rhenium analogue showed relatively low efficiency, although the reactivity could be enhanced minimally by increasing the temperature or changing the oxygen source from LiClO_4_ to AgClO_4_. Notably, the activity trend of the three rhenium complexes reversed when using the typical DMSO/PPh_3_ system with the highest activity for the most rigid ligand backbone, whereas the *cis*-complex with the most flexible backbone was completely inactive [26].

Evaluation of the bioinspired molybdenum-based functional analogues with PymS and PyS ligands implied full reduction of perchlorate to chloride, which was quantified indirectly by monitoring the OPPh_3_ concentration and directly by determination of the perchlorate and chloride concentration with a new high-performance liquid chromatography-inductively coupled plasma mass spectrometry (HPLC-ICPMS/MS) method [135,139]. The catalytic rate for the molybdenum complex bearing the PymS ligand was slightly enhanced, retaining even activity at decreased catalyst load. Still, the TON of the [ReOCl(hoz)_2_] complex from Abu-Omar and others was found to be 31 after 4 h compared to a TON of 30 after 24 h for [MoO_2_(PymS)_2_] [130]. During catalysis, no intermediate chlorate, chlorite, or hypochlorite was detected, presumably due to the high reactivity of these species. This observation was essentially confirmed in the absence of an oxygen acceptor with the one way reaction with (per)chlorate which only led to different detected side products, such as a µ-oxido dimer, a tetrakis(PymS) complex or the ligand disulfide (PymS)_2_ [135]. It must be pointed out though, that these bioinspired molybdenum complexes represent the first promising molybdenum-containing functional analogues capable of homogenously mimicking the natural perchlorate reduction.

**Figure 12:**
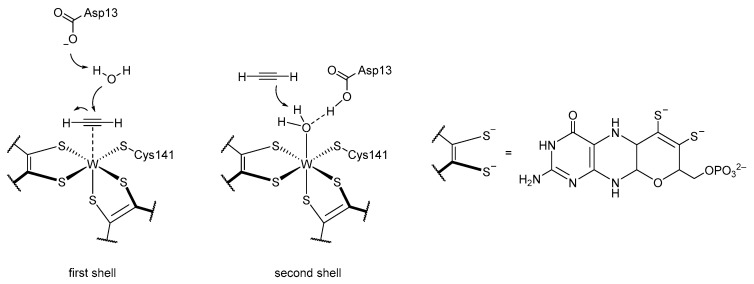
Comparison of the first shell (**left**) and second shell mechanism (**middle**) regarding the binding mode of the substrate to the active site of acetylene hydratase. MPT (**right**) is shown as pruned on the left. Adapted from the literature [147].

Among the molybdo- and tungstoenzymes, the tungsten-dependent acetylene hydratase comprises a rather unique type resulting in it being the sole member of its own family according to the Hille classification (see Figure 2) [6]. The catalyzed reaction is quite unusual for an oxidoreductase, because the substrate acetylene undergoes hydration to yield acetaldehyde. To date, the reaction mechanism is not unraveled, but two general reaction pathways are discussed regarding the mode of substrate binding (Figure 12). The first pathway proposed by Liao and others, also called “first shell mechanism”, comprises replacing water by acetylene in the first coordination sphere, followed by a nucleophilic attack of the liberated water molecule interacting with an aspartate residue and the acetylene in the second coordination sphere resulting in acetaldehyde at the activated acetylene [140,141,142]. The second pathway proposed by Einsle and others, also called “second shell mechanism,” assumes a direct nucleophilic attack of the water while it is still coordinated to tungsten, whereas the acetylene substrate is located in the second coordination sphere [143]. Some structural analogues can be found in the literature, which mimic the binding of acetylene to tungsten centers [144,145,146].

Modelling the function of acetylene hydratase represents a challenging task and the literature lacks functional analogues. Only one system was reported in 1997 as an active model complex by Sarkar and others: the tungsten dithiolene complex (Et_4_N)_2_[WO(mnt)_2_] [148,149]. However, later theoretical and experimental investigations challenged this report and are in accordance with the fact that no intermediate or acetylene adduct was isolable [150].

**Figure 13:**
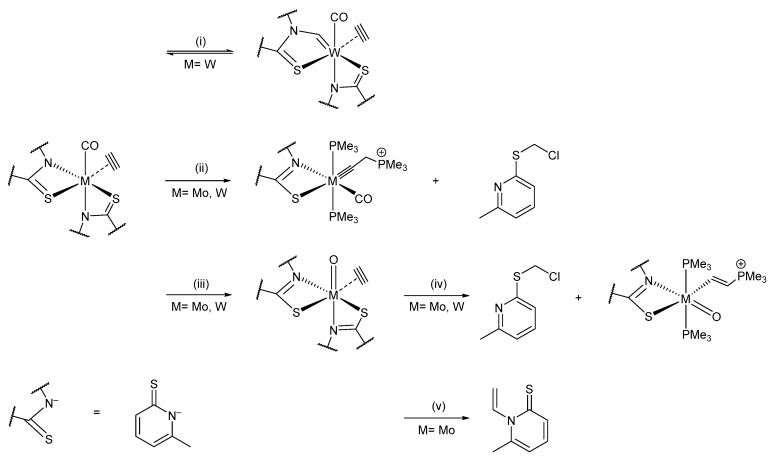
Reactivity of model complexes [M^II^(CO)(C_2_H_2_)(6-MePyS)_2_] (M=Mo, W; 6-MePyS = 6-methylpyridine-2-thiolate) exhibiting acetylene activation; (i) CH_2_Cl_2_, under C_2_H_2_ atmosphere, 24 h; (ii) 3 eq. PMe_3_, CH_2_Cl_2_, 4 h; (iii) M = Mo, 1.15 eq. Me_3_NO, CH_2_Cl_2_, 0 °C, 1.5 h; M = W, 1.15 eq. pyridine-N-oxide, CH_2_Cl_2_, 20 h; (iv) M = Mo, 3 eq. PMe_3_, CH_2_Cl_2_, 1.5 h; M = W, 3 eq. PMe_3_, CH_2_Cl_2_, 7 h; (v) 5 eq. H_2_O, 5 eq. Et_3_N, CH_3_CN, 15 h [151,152,153].

More recently, extensive functional studies were carried out by the group of Mösch-Zanetti employing the [W^II^(CO)(C_2_H_2_)(PyS)_2_] complexes with different PyS derivatives (Figure 13) [147,151,152,153]. The corresponding complex [W^IV^O(C_2_H_2_)(PyS)_2_] was synthesized with oxygen donor reagent pyridine-N-oxide (PyNO) to establish a structurally more similar analogue of the reduced active site. With an excess of C_2_H_2_, the authors showed the first nucleophilic attack on acetylene bound to a mononuclear tungsten complex [W^II^(CO)(C_2_H_2_)(PyS)_2_], evidenced by insertion of acetylene into the W-N coordinative bond of one ligand to yield a [W(CO)(C_2_H_2_)(CHCH-PyS)(PyS)] complex [147,151]. Further investigations focused on using PMe_3_ as a nucleophile to activate the acetylene bond at the W^II^ and W^IV^ species while using a methylated PyS ligand to prevent the C_2_H_2_ insertion into the W-N bond [153]. As a result, in both cases, a 2((chloromethyl)thio)-pyridine adduct was observed resulting from the fusion of one PyS ligand and dichloromethane. However, acetylene behaved differently depending on the formal oxidation state of tungsten. In the case of W^II^, acetylene and PMe_3_ generated a P-C bond accompanied by formation of an ethylidene, whereas for the W^IV^ complex the same P-C bond formation was observed accompanied by ethenyl generation. The reactivity study was extended to molybdenum with the [Mo^IV^O(C_2_H_2_)(PyS)_2_] complex [152]. This compound reacted with excess of acetylene and traces of water yielding the N-vinylated 1-vinylpyridine-2(1H)-thione which is the protonated CHCH-PyS species formerly reported for the W^II^ analogue. In addition, the ethenyl species plus the 2-thiopyridine were observed with PMe_3_ as nucleophile in dichloromethane. This research facilitated some first and very valuable insights into the activation of acetylene when coordinated to a mononuclear tungsten or molybdenum complex, thereby at least tentatively supporting the first shell mechanism.

## 3. Functional Dithiolene-Bearing Analogues with Atypical/Non-Natural Reactivity

Research into the coordination chemistry with dithiolene ligands started in the 1930s with toluene-3,4-dithiol and 1-chlorobenzene-3,4-dithiol [154,155,156]. A milestone was the discovery of dithiolenes’ *non-innocent* character and its impact on the electronic properties of the coordinated metal centers in the 1960s (see Figure 1 above), which was first noted for nickel bis(dithiolene) complexes [3,157,158,159,160]. In the late 1980s, the detection of the dithiolene moiety as component of the MPT ligand unravelled molybdopterin’s important role for enzyme reactivity by modulating the electronic structure of the active site [11,161,162]. As a result, enzymologists and coordination chemists began combining their efforts to decipher the electronic and chemical structures, reactivities and catalytic mechanisms of the active sites in mononuclear molybdenum and tungsten-dependent enzymes. Dithiolene chemistry was never restricted to biological issues, though, and these fascinating ligands and their coordination compounds continued to be extensively used for chemical purposes, and in particular for reactions in which their special redox modulating properties were anticipated or found to be advantageous. Consequently, dithiolene compounds are widely used with quite some versatility for often interdisciplinary scientific research topics, such as conducting materials, magnetism, MOF chemistry (including small molecule adsorption), gas sensor technique, or optical devices [163,164,165,166,167,168,169].

Considering the scope and the bioinorganic chemical context of this review, their use in homogenous catalysis will constitute the focus of this last section (i.e., excluding heterogeneous applications). In addition, a plethora of reports with a broad range of different coordinated metal centers is available in the literature. In the following, the emphasis is on homogenous catalysis and uncommon non-catalytic reactivity with predominantly molybdenum and tungsten coordination centers bearing biologically inspired ligand scaffolds (dithiolenes) in continuation of the previous two sections (*vide supra*).

### 3.1. Catalytic Hydrogen Evolution Reaction (HER)

The acceptance that climate change poses already in some parts of the earth a life-threatening and universally ever-growing crisis results in steadily rising efforts to convert the global energy sector. Eco-friendly hydrogen production has become a center of attention in this respect and is believed to be, among others, a probable solution for energy storage and distribution. One option in this regard is the utilization of homogenous transition metal catalysts coordinated, for instance, by *non-innocent* dithiolene ligands. The dithiolene ligands are presumed to participate in some extended electron relay events, thereby supporting the redox activity at the metal center [169]. Fontecave and others introduced several bioinspired molybdenum, tungsten- and cobalt-dependent dithiolene (qpdt, dmed, mnt) complexes, which exhibit electrocatalytic and photocatalytic activity toward hydrogen production (already discussed above as unwanted side reactions) [78,170,171,172].

**Figure 14:**
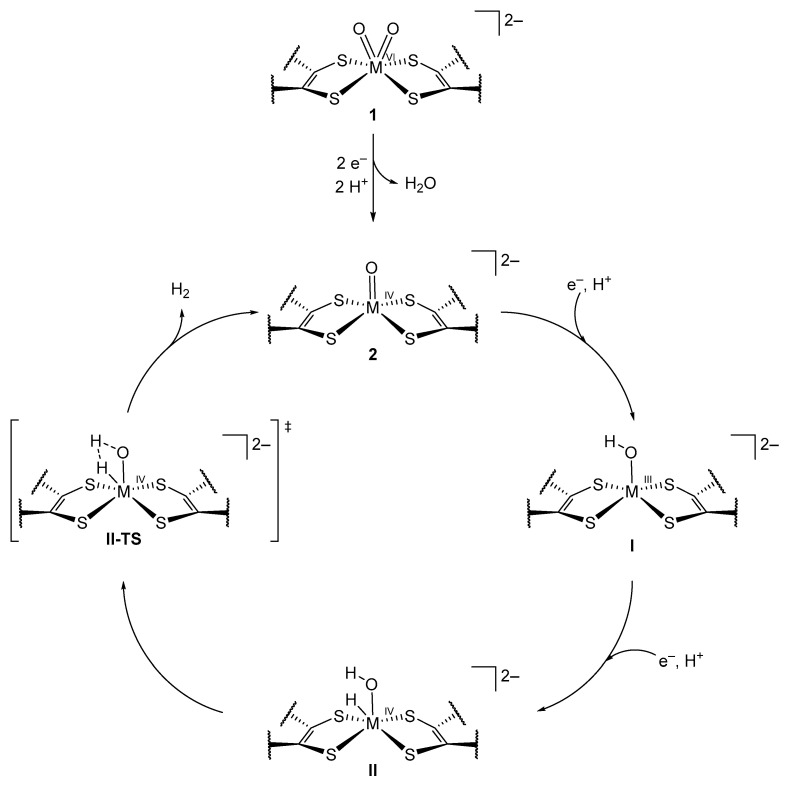
Proposed catalytic mechanism for the hydrogen evolution reaction mediated by a [M^VI^O_2_(dt)_2_]^2−^ (1); M = W, Mo; dt = dithiolene) or [M^IV^O(dt)_2_]^2−^ (2) complex catalyst. 1 represents the pre-catalyst which is converted into the active catalyst 2. I and II are non-isolable intermediates during the catalytic cycle yielding the transition state II-TS which releases hydrogen when transforming back to 1. Adapted from the original literature reports [78,170].

The complex salts (Et_4_N)_2_[Mo^IV^O(qpdt)_2_] and (Et_4_N)_2_[Co^III^(qpdt)_2_]_2_ were found to be relatively good mimics of the active site of mononuclear molybdenum enzymes considering their MPT-resembling ligand system. They were additionally investigated for their ability to mediate the reduction of protons to molecular hydrogen. TOF values were determined for the electrocatalytic (1030 s^−1^ (Mo, 0.1 M trifluoroacetic acid) vs. 5570 s^−1^ (Co, 0.1 M acetic acid)) and photocatalytic hydrogen evolution (Initial TOF: 203 h^−1^ (Mo) vs. 163 h^−1^ (Co)) with a notable difference in activity. Theoretical studies were carried out in order to support the experimental data and to understand the nature of this reaction. The computations revealed the importance of a protonation at one of the pyrazine nitrogen atoms (the one not adjacent to the pyrene oxygen) in the qpdt ligand (Figure 14). For the cobalt species, hydrogen evolution was apparently induced by a metal-bonded hydride interacting with a nearby proton, either as part of a hydroxide ligand on molybdenum or in form of a protonated sulfur atom of the dithiolene coordination site [78,172].

Bioinspired tungsten catalysts [W^VI^O_2_(dt)_2_]^2−^ (dt = dmed, mnt) were used for the same purpose of hydrogen evolution. In contrast to the previous examples, strongly electron-withdrawing dithiolene ligands with only minimal similarity to natural MPT were chosen. These complexes were also able to produce hydrogen via electrocatalysis and photocatalysis; however, their lack in efficiency was substantial compared to the molybdenum and cobalt examples (Table 1). Theoretical calculations and reactivity studies with [W^IV^O(dmed)_2_]^2−^ corroborated the catalytic mechanism which was already identified for the molybdenum complex with qpdt comprising a transition state with a metal-hydride species that interacts with the adjacent proton of a hydroxide ligand (Figure 14) [170].

The involvement of a molybdenum-hydride species had already been predicted by the group of Eisenberg in 2014 with regard to the hydrogen evolution reaction being mediated by desoxo Mo^IV^ complexes utilizing dithiolene ligands tdt, bdt and 3,6-chloro-benzen-1,2-dithiolate (bdtCl_2_) with isocyanide or phosphane co-ligands [173]. The complexes, upon 2-electron reduction, released the co-ligand with subsequent addition of aqueous protons forming the hydride species; this then yielded molecular hydrogen after contact with a proton.

Another desoxido W^IV^ complex was reported to produce hydrogen after addition of trace amounts of water under CO_2_ atmosphere leading to a monooxido W^V^ species and hydrogen [174]. Intriguing as this observation is, it requires further investigation before any more general conclusions can be drawn from it.

### 3.2. Non-Catalytic Reactivity toward Small Molecules

**Figure 15:**
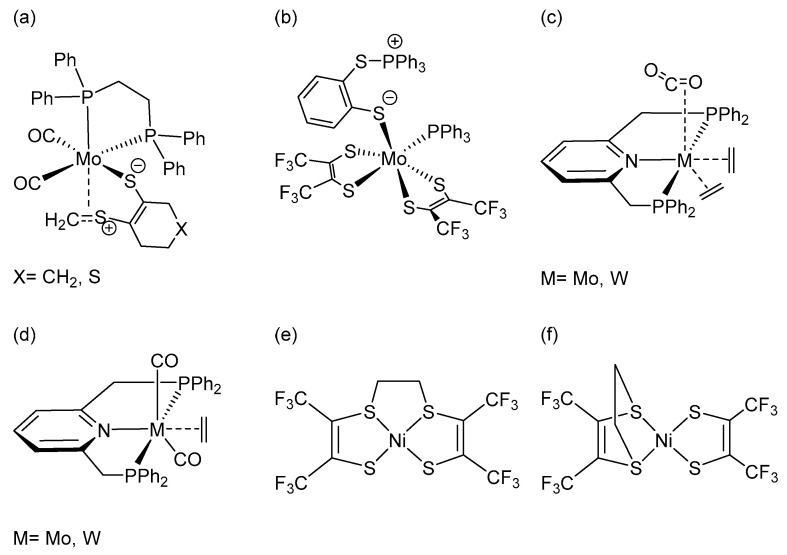
Selected non-natural reaction products associated with functional analogues of molybdenum and tungsten-dependent oxidoreductases (**a**–**d**). (**a**) [Mo(CO)_2_(CH_2_-dt)(dppe)] (dt: cydt = cyclohex-1-ene-1,2-dithiolate or tpydt = 5,6-dihydro-2H-thiopyran-3,4-dithiolate; dppe = 1,2-bis(diphenylphosphino)ethane) [36], (**b**) [Mo(tfd)_2_(SC_6_H_4_SPPh_3_)(PPh_3_)] [176], (**c**) [M(C_2_H_4_)_2_(CO_2_)(PNP)] (M = Mo, W; PNP = 2,6-bis(diphenylphosphinomethyl)pyridine) [177], (**d**) [M(C_2_H_4_)(CO)_2_(PNP)] [177] (**e**,**f**) Two possible binding modes of ethylene to [Ni(tfd)_2_] (tfd = 1,2-trifluoromethyl-1,2-dithiolate) [169,175,178].

(Bio-)inorganic chemists on their quest towards unravelling the secrets of molybdenum and tungsten dithiolene chemistry, have to be aware that they may encounter unpredicted and rather surprising reactivities going back to the *non-innocence* character of the dithiolene. The inherent sulfur-based nucleophilic potential of the ligand can lead to a ligand-based, instead of a metal-based, reactivity [36,169,175]. Elvers et al., when examining the molybdenum mono(dithiolene) complex [Mo(CO)_2_(dt)(dppe)] (dt: cydt = cyclohex-1-ene-1,2-dithiolate or tpydt = 5,6-dihydro-2H-thiopyran-3,4-dithiolate; dppe = 1,2-bis(diphenylphosphino)ethane), noted and proved an unforeseen reactivity towards dichloromethane solvent upon electrochemical 2-electron reduction. The resulting derivatized complex [Mo(CO)_2_(CH_2_-dt)(dppe)] could be isolated and structurally characterized. It is the result of the unexpected formation of a sulfur-alkylated sulfonium species which π-coordinates the molybdenum center via its methylene bond (Figure 15a). This reaction product represents a rather rare case of a positively charged ligand bound to a molecular complex [36].

Although this was the very first example of an S-alkylated sulfonium dithiolene, the underlying chemistry is not that much different from the addition of PPh_3_ to the tris(dithiolene) complex [Mo(tfd)2(bdt)] (tfd = 1,2-trifluoromethyl-1,2-ethylendithiolate) resulting in the formation of an S-P bond, which gives a monodentate zwitterionic SC_6_H_4_SPPh_3_ adduct [176]. The corresponding molybdenum complex [Mo(tfd)_2_(SC_6_H_4_SPPh_3_)(PPh_3_)] was isolated and comprehensively characterized (Figure 15b). In both cases it is undisputed that the transformations are mediated by the central molybdenum in the precursor complexes.

The sulfur-centered nucleophilic character of the dithiolene ligand can also support the reversible binding of olefins. In the process, the olefin double bond is broken and the reaction is reminiscent of typical cycloadditions (Figure 15e,f) [169,175]. The first examples of olefin reactivity were reported for nickel bis(dithiolene) complexes and strained (hence pre-activated) cyclic alkenes were applied [179,180,181]. Subsequently, researchers targeted the more challenging activation of simple olefins, including ethylene, with transition metal dithiolene complexes, even linking this approach to developing new purification methods for olefins from petrochemical feedstocks [169,178,182]. The normally very unreactive molybdenum tris(dithiolene) complexes were, in this context, used by Fekl et al., who could initiate and observe binding of ethylene to a push-pull molybdenum complex [175]. However, most of the recent respective research activities focused on theoretical calculations of the binding properties and ligand impact on the addition of olefins; there is, thus, a lack in topical experimental data [182,183,184,185,186,187]. Possibly inspired by the success with the dithiolene systems, Carmona et al. applied an alternative coordination environment; they developed molybdenum and tungsten complexes with a pyridine-based pincer ligand 2,6-bis(diphenylphosphinomethyl)pyridine (PNP) and examined ethylene addition, resulting in an ethylene rich complex [M(C_2_H_4_)_3_(PNP)] (M = Mo, W). Interestingly, one or two ethylene groups were replaceable by CO_2_ and CO, respectively, revealing a system which was assumed to be relevant for catalytic acrylate formation (Figure 15c,d) [177].

## 4. Conclusions

Throughout the last decade the field of bioinorganic functional model chemistry targeting molybdenum and tungsten-dependent oxidoreductases has witnessed many, substantially varied developments. The respective scientific community appears not to be as busy as before, but the findings it has facilitated lately still have enormous relevance and often come with elements of surprise.

Modelling biological oxygen atom transfer catalysis with molybdenum and tungsten complexes carrying dithiolene ligands represents and remains a daring task. Understanding mechanistic details of the half cycle transformations has improved substantially within the last ten years with the help of dithiolene complex chemistry regarding both, OAT (from and to metal ion centers) and PCET. Efficacy can be modulated by electronic effects and the interplay of ligand and metal, resulting in reaction rate changes. Trends in rate constants for chalcogenide atom transfer could be derived (k_Se_ > k_S_ > k_O_). Differences were observed for the oxygen atom donation ability of molybdenum and tungsten (generally but not exclusively: k_Mo_ > k_W_). Using electrochemical or photochemical approaches facilitated even more advanced studies of the reactivity of suitable catalysts or pre-catalysts as functional analogues for molybdenum- and tungsten-containing oxidoreductases, with which also unusual transformations (CO, H_2_) could be targeted.

Non-dithiolene model chemistry enables the synthesis of new functional analogues for different mononuclear molybdenum or tungsten-based enzymes, including those with uncommon reactivities and challenging substrates. Variations in the metal center as well as of the ligand environment helped unravelling electronic preferences of oxygen atom transfer reactions and support observations made with biologically relevant dithiolene complexes. Notable natural reactivities, such as perchlorate and nitrate reduction as well as the activation of acetylene, can be visualized in a wide range of applications, though the respective model chemistry is difficult, rendering the recent respective success with molybdenum and tungsten coordination chemistry ever more valuable.

Surprising non-biological transformations were observed with dithiolene complexes, in particular with more ‘extreme’ oxidation states of the central metal and those resulting in modifications of the ligand rather than in interactions with the metal center. Findings in this respect are most often serendipitous.

Respective research throughout the last decade has substantially impacted knowledge and even potential applications. Still, many scientific issues have not been entirely resolved, as of yet. In particular, with regard to the PCET half of the natural catalytic cycles, there are still many intricate issues needing to be addressed. The development of suitable systems (model complexes, purely aqueous conditions, controlled pH values, controlled proton donors/acceptors), at the same time, remains exceptionally challenging. In addition, the enzymes’ substrate/reaction diversity as we know it may not be at its limits as of yet, considering that possibly enzymes are still waiting to be discovered.

Bioinorganic chemists working in this field will hardly run out of important topics to be tackled anytime soon. The molybdenum and tungsten oxidoreductases will continue surprising, intriguing, and fascinating us—for at least another decade by our prediction.

## Figures and Tables

**Scheme 1:** Anaerobic bacterial respiration of perchlorate, decomposing perchlorate to chloride enabled by perchlorate reductase (PcrAB) and chlorite dismutase (Cld) [122].

$${ClO}_{4}^{-}\overset{\text{ PcrAB }}{\to }{ClO}_{3}^{-}\overset{\text{ PcrAB }}{\to }{ClO}_{2}^{-}\overset{Cld}{\to }{Cl}^{-}+O_{2}$$

**Scheme 2:** Different metabolic pathways for nitrate: (**top**) typical denitrification, (**middle**) dissimilatory nitrate reduction to ammonium, (**bottom**) anaerobic ammonium oxidation (Anammox); NarG: membrane-bound nitrate reductase, NirSK: nitrite reductase, NOR: nitric oxide reductase, NOS: nitrous oxide reductase, NapAB: periplasmic nitrate reductase, NrfA: nitrite reductase, ONR: octaheme cytochrome c reductase, HH: hydrazine hydrolase, HZO: hydrazine-oxidizing enzyme, HAO: hydrazine oxidoreductase [124].

$$\begin{array}{c} {NO}_{3}^{-}\overset{NarG}{\to }{NO}_{2}^{-}\overset{NirSK}{\to }NO\overset{NOR}{\to }N_{2}O\overset{NOS}{\to }N_{2}\\ {NO}_{3}^{-}\overset{NapAB}{\to }{NO}_{2}^{-}\overset{NrfA/ONR}{\to }{NH}_{4}^{+}\\ {NO}_{3}^{-}\overset{NarG}{\to }{NO}_{2}^{-}\overset{\text{ NirSK }}{\to }NO\overset{\underset{HH}{+{NH}_{4}^{+}}}{\to }N_{2}H_{4}\overset{HZO/HAO}{\to }N_{2} \end{array}$$

**Table 1:** Comparison of turnover frequency (TOF) values for the hydrogen evolution reaction via photocatalysis and electrocatalysis evaluated for different functional analogues [78,170,172].

TOF	[MoO(qpdt)_2_]^2−^	[Co(qpdt)_2_]_2_^2−^	[WO_2_(mnt)_2_]^2−^	[WO_2_(dmed)_2_]^2−^
photocatalysis ^1^ [h^−1^]	203	163	12	4
electrocatalysis [s^−1^]	1030	5570	143	63

^1^ Data represent initial TOF values.

## References

[1] Majumdar A., Sarkar S. (2011). Bioinorganic chemistry of molybdenum and tungsten enzymes: A structural-functional modeling approach. Coord. Chem. Rev..

[2] Hine F.J., Taylor A.J., Garner C.D. (2010). Dithiolene complexes and the nature of molybdopterin. Coord. Chem. Rev..

[3] Eisenberg R., Gray H.B. (2011). Noninnocence in Metal Complexes: A Dithiolene Dawn. Inorg. Chem..

[4] Stiefel E.I. (2003). Dithiolene Chemistry: Synthesis, Properties, and Applications.

[5] Hille R. (1996). The Mononuclear Molybdenum Enzymes. Chem. Rev..

[6] Hille R. (2002). Molybdenum and tungsten in biology. Trends Biochem. Sci..

[7] Romao M.J. (2009). Molybdenum and tungsten enzymes: A crystallographic and mechanistic overview. Dalton Trans..

[8] Holm R.H., Solomon E.I., Majumdar A., Tenderholt A. (2011). Comparative molecular chemistry of molybdenum and tungsten and its relation to hydroxylase and oxotransferase enzymes. Coord. Chem. Rev..

[9] Schulzke C. (2011). Molybdenum and Tungsten Oxidoreductase Models. Eur. J. Inorg. Chem..

[10] Enemark J., Cooney J.J., Wang J.J., Holm R.H. (2004). Synthetic Analogues and Reaction Systems Relevant to the Molybdenum and Tungsten Oxotransferases. Chem. Rev..

[11] Holm R.H. (1990). The biologcally relevant oxygen atom transfer chemistry of molybdenum: From synthetic analogue systems to enzymes. Coord. Chem. Rev..

[12] Kaim W., Schwederski B. (2005). Bioanorganische Chemie.

[13] Arumuganathan T., Volpe M., Harum B., Wurm D., Belaj F., Mosch-Zanetti N.C. (2012). Unusual nonoctahedral geometry with molybdenum oxoimido complexes containing eta2-pyrazolate ligands. Inorg. Chem..

[14] Volpe M., Mösch-Zanetti N.C. (2012). Molybdenum(VI) Dioxo and Oxo-Imido Complexes of Fluorinated β-Ketiminato Ligands and Their Use in OAT Reactions. Inorg. Chem..

[15] Ehweiner M.A., Wiedemaier F., Belaj F., Mösch-Zanetti N.C. (2020). Oxygen Atom Transfer Reactivity of Molybdenum(VI) Complexes Employing Pyrimidine- and Pyridine-2-thiolate Ligands. Inorg. Chem..

[16] Peschel L., Vidovic C., Belaj F., Neshchadin D., Mösch-Zanetti N. (2019). Activation and Photoinduced Release of Alkynes on a Biomimetic Tungsten Center: The Photochemical Behavior of the W−S-Phoz System. Chem. Eur. J..

[17] Leppin J., Förster C., Heinze K. (2014). Molybdenum Complex with Bulky Chelates as a Functional Model for Molybdenum Oxidases. Inorg. Chem..

[18] Hüttinger K., Förster C., Heinze K. (2014). Intramolecular electron transfer between molybdenum and iron mimicking bacterial sulphite dehydrogenase. Chem. Commun..

[19] Tran B.L., Arita A., Cooksy A.L., Carrano C.J. (2016). Examination of oxygen atom transfer reactivity of heteroscorpionate dioxo-Mo(VI) complexes: Geometric isomers, solvent effect, intermediates, and catalytic oxidation. Inorg. Chim. Acta.

[20] Ma X., Schulzke C. (2013). Molybdenum and tungsten complexes of bis (phenolate) ligands, O, X, O (X = S or Se): Synthesis, characterization and catalytic oxygen atom transfer properties. Inorg. Chim. Acta.

[21] Arumuganathan T., Mayilmurugan R., Volpe M., Mosch-Zanetti N.C. (2011). Faster oxygen atom transfer catalysis with a tungsten dioxo complex than with its molybdenum analog. Dalton Trans..

[22] Mayilmurugan R., Harum B.N., Volpe M., Sax A.F., Palaniandavar M., Mosch-Zanetti N.C. (2011). Mechanistic insight into the reactivity of oxotransferases by novel asymmetric dioxomolybdenum(VI) model complexes. Chem. Eur. J..

[23] Schindler T., Sauer A., Spaniol T.P., Okuda J. (2018). Oxygen Atom Transfer Reactions with Molybdenum Cofactor Model Complexes That Contain a Tetradentate OSSO-Type Bis(phenolato) Ligand. Organometallics.

[24] Maurya M.R., Uprety B., Avecilla F. (2016). Dioxidomolybdenum(VI) Complexes of Tripodal Tetradentate Ligands for Catalytic Oxygen Atom Transfer between Benzoin and Dimethyl Sulfoxide and for Oxidation of Pyrogallol. Eur. J. Inorg. Chem..

[25] Schachner J.A., Terfassa B., Peschel L.M., Zwettler N., Belaj F., Cias P., Gescheidt G., Mosch-Zanetti N.C. (2014). Oxorhenium(V) complexes with phenolate-oxazoline ligands: Influence of the isomeric form on the O-atom-transfer reactivity. Inorg. Chem..

[26] Zwettler N., Schachner J.A., Belaj F., Mösch-Zanetti N.C. (2016). Oxidorhenium(V) Complexes with Tetradentate Iminophenolate Ligands: Influence of Ligand Flexibility on the Coordination Motif and Oxygen-Atom-Transfer Activity. Inorg. Chem..

[27] Chrysochos N., Ahmadi M., Wahlefeld S., Rippers Y., Zebger I., Mroginski M.A., Schulzke C. (2019). Comparison of molybdenum and rhenium oxo bis-pyrazine-dithiolene complexes–in search of an alternative metal centre for molybdenum cofactor models. Dalton Trans..

[28] Lohrey T.D., Bergman R.G., Arnold J. (2016). Oxygen Atom Transfer and Intramolecular Nitrene Transfer in a Rhenium β-Diketiminate Complex. Inorg. Chem..

[29] Lohrey T.D., Cortes E.A., Fostvedt J.I., Oanta A.K., Jain A., Bergman R.G., Arnold J. (2020). Diverse Reactivity of a Rhenium(V) Oxo Imido Complex: [2 + 2] Cycloadditions, Chalcogen Metathesis, Oxygen Atom Transfer, and Protic and Hydridic 1,2-Additions. Inorg. Chem..

[30] Moula G., Bose M., Sarkar S. (2013). Replica of a fishy enzyme: Structure–function analogue of trimethylamine-N-oxide reductase. Inorg. Chem..

[31] Ghosh A.C., Samuel P.P., Schulzke C. (2017). Synthesis, characterization and oxygen atom transfer reactivity of a pair of Mo (iv) O-and Mo (vi) O 2-enedithiolate complexes–a look at both ends of the catalytic transformation. Dalton Trans..

[32] Ahmadi M., Fischer C., Ghosh A.C., Schulzke C. (2019). An Asymmetrically Substituted Aliphatic Bis-Dithiolene Mono-Oxido Molybdenum(IV) Complex With Ester and Alcohol Functions as Structural and Functional Active Site Model of Molybdoenzymes. Front. Chem..

[33] Sugimoto H., Sato M., Asano K., Suzuki T., Ogura T., Itoh S. (2019). Oxido-alcoholato/thiolato-molybdenum(VI) complexes with a dithiolene ligand generated by oxygen atom transfer to the molybdenum(IV) complexes. Inorg. Chim. Acta.

[34] Dille S.A., Colston K.J., Mogesa B., Cassell J., Perera E., Zeller M., Basu P. (2021). The Impact of Ligand Oxidation State and Fold Angle on the Charge Transfer Processes of Mo IV O-Dithione Complexes. Eur. J. Inorg. Chem..

[35] Elvers B.J., Schulzke C., Fischer C. (2019). Photochemical Unmasking of 1,3-Dithiol-2-ones: An Alternative Route to Heteroleptic Dithiolene Complexes from Low-Valent Molybdenum and Tungsten Precursors. Eur. J. Inorg. Chem..

[36] Elvers B.J., Krewald V., Schulzke C., Fischer C. (2021). Reduction induced S-nucleophilicity in mono-dithiolene molybdenum complexes–in situ generation of sulfonium ligands. Chem. Commun..

[37] Holm R.H. (1987). Metal-centered oxygen atom transfer reactions. Chem. Rev..

[38] Fischer C., Fischer L., Hille R., Schulzke C., Kirk M.L. (2016). Comparitive Kinetics of Enzymes and Models. Molybdenum and Tungsten Enzymes: Bioinorganic Chemistry.

[39] Hille R., Schulzke C., Kirk M.L. (2016). Molybdenum and Tungsten Enzymes: Bioinorganic Chemistry.

[40] Garton S.D., Hilton J., Oku H., Crouse B.R., Rajagopalan K.V., Johnson M.K. (1997). Active Site Structures and Catalytic Mechanism of Rhodobacter sphaeroides Dimethyl Sulfoxide Reductase as Revealed by Resonance Raman Spectroscopy. J. Am. Chem. Soc..

[41] Webster C.E., Hall M.B. (2001). The Theoretical Transition State Structure of a Model Complex Bears a Striking Resemblance to the Active Site Structure of DMSO Reductase. J. Am. Chem. Soc..

[42] Schindelin H., Kisker C., Hilton J., Rajagopalan K.V., Rees D.C. (1996). Crystal Structure of DMSO Reductase: Redox-Linked Changes in Molybdopterin Coordination. Science.

[43] Hilton J., Rajagopalan K.V. (1996). Identification of the Molybdenum Cofactor of Dimethyl Sulfoxide Reductase fromRhodobacter sphaeroidesf. sp.denitrificansas Bis(molybdopterin guanine dinucleotide)molybdenum. Arch. Biochem. Biophys..

[44] Ha Y., Tenderholt A.L., Holm R.H., Hedman B., Hodgson K.O., Solomon E.I. (2014). Sulfur K-edge X-ray Absorption Spectroscopy and Density Functional Theory Calculations on Monooxo Mo^IV^ and Bisoxo Mo^VI^ Bis-dithiolenes: Insights into the Mechanism of Oxo Transfer in Sulfite Oxidase and Its Relation to the Mechanism of DMSO Reductase. J. Am. Chem. Soc..

[45] Sugimoto H., Sato M., Asano K., Suzuki T., Mieda K., Ogura T., Matsumoto T., Giles L.J., Pokhrel A., Kirk M.L. (2016). A Model for the Active-Site Formation Process in DMSO Reductase Family Molybdenum Enzymes Involving Oxido−Alcoholato and Oxido−Thiolato Molybdenum(VI) Core Structures. Inorg. Chem..

[46] Sugimoto H., Hatakeda K., Toyota K., Tatemoto S., Kubo M., Ogura T., Itoh S. (2013). A new series of bis(ene-1,2-dithiolato)tungsten(IV), -(V), -(VI) complexes as reaction centre models of tungsten enzymes: Preparation, crystal structures and spectroscopic properties. Dalton Trans..

[47] Sugimoto H., Sato M., Giles L.J., Asano K., Suzuki T., Kirk M.L., Itoh S. (2013). Oxo-carboxylato-molybdenum(vi) complexes possessing dithiolene ligands related to the active site of type II DMSOR family molybdoenzymes. Dalton Trans..

[48] Sugimoto H., Tano H., Miyake H., Itoh S. (2011). Generation of bis(dithiolene)dioxomolybdenum(VI) complexes from bis(dithiolene)monooxomolybdenum(IV) complexes by proton-coupled electron transfer in aqueous media. Dalton Trans..

[49] Sugimoto H., Tano H., Suyama K., Kobayashi T., Miyake H., Itoh S., Mtei R.P., Kirk M.L. (2011). Chalcogenidobis(ene-1,2-dithiolate)molybdenum(IV) complexes (chalcogenide E = O, S, Se): Probing Mo identical withE and ene-1,2-dithiolate substituent effects on geometric and electronic structure. Dalton Trans..

[50] Sugimoto H., Tatemoto S., Toyota K., Ashikari K., Kubo M., Ogura T., Itoh S. (2013). Oxo-sulfido- and oxo-selenido-molybdenum(vi) complexes possessing a dithiolene ligand related to the active sites of hydroxylases of molybdoenzymes: Low temperature preparation and characterisation. Chem. Commun..

[51] Hasenaka Y., Okamura T.-a., Onitsuka K. (2015). Efficient uptake of dimethyl sulfoxide by the desoxomolybdenum(iv) dithiolate complex containing bulky hydrophobic groups. Dalton Trans..

[52] Okamura T.-a., Ushijima Y., Omi Y., Onitsuka K. (2012). Systematic Investigation of Relationship between Strength of NH···S Hydrogen Bond and Reactivity of Molybdoenzyme Models. Inorg. Chem..

[53] Baba K., Okamura T., Suzuki C., Yamamoto H., Yamamoto T., Ohama M., Ueyama N. (2006). O-Atom-Transfer Oxidation of [Molybdenum(IV) Oxo{3,6-(acylamino) 2 -1,2-benzenedithiolato}2] 2- Promoted by Intramolecular NH‚‚‚S Hydrogen Bonds. Inorg. Chem..

[54] Swenson D., Baenziger N.C., Coucouvanis D. (1978). Tetrahedral mercaptide complexes. Crystal and molecular structures of [(C6H5)4P]2M(SC6H5)4 complexes (M = cadmium(II), zinc(II), nickel(II), cobalt(II), and manganese(II)). J. Am. Chem. Soc..

[55] Okamura T., Takamizawa S., Ueyama N., Nakamura A. (1998). Novel Rubredoxin Model Tetrathiolato Iron(II) and Cobalt(II) Complexes Containing Intramolecular Single and Double NH···S Hydrogen Bonds. Inorg. Chem..

[56] Garner C.D., Nicholson J.R., Clegg W. (1984). Preparation, crystal structure, and spectroscopic characterization of [NEt4]2[Cu(SPh)3]. Inorg. Chem..

[57] Okamura T., Ueyama N., Nakamura A., Ainscough E.W., Brodie A.M., Waters J.M. (1993). The effect of strong NH...S hydrogen bonds in the copper(I) thiolate complex,(NEt4)2[Cu(o-pabt)3](o-pabt =o-pivaloylaminobenzenethiolato). J. Chem. Soc. Chem. Commun..

[58] Boyde S., Ellis S.R., Garner C.D., Clegg W. (1986). Structural Comparison of Oxobis(benzene-l,2-dithiolato)molybdenum-(v) and -(Iv) Complexes. J. Chem. Soc. Chem. Commun..

[59] Baba K., Okamura T., Yamamoto H., Yamamoto T., Ohama M., Ueyama N. (2006). Dioxotungsten 1,2-Benzenedithiolate Complex Stabilized by NH--S Hydrogen Bonds. Inorg. Chem..

[60] Okamura T.A., Tatsumi M., Omi Y., Yamamoto H., Onitsuka K. (2012). Selective and effective stabilization of Mo(VI) horizontal lineO bonds by NH...S hydrogen bonds via trans influence. Inorg. Chem..

[61] Moura J.J.G., Brondino C.D., Trincao J., Romao M.J. (2004). Mo and W bis-MGD enzymes: Nitrate reductases and formate dehydrogenases. J. Biol. Inorg. Chem..

[62] Campbell W.H. (1999). Nitrate Rreductase Structure, Function and Regulation: Bridging the Gap between Biochemistry and Physiology. Annu. Rev. Plant Physiol. Plant Mol. Biol..

[63] Wang J.J., Tessier C., Holm R.H. (2006). Analogue Reaction Systems of Selenate Reductase. Inorg. Chem..

[64] Lim B.S., Donahue J.P., Holm R.H. (2000). Synthesis and Structures of Bis(dithiolene)molybdenum Complexes Related to the Active Sites of the DMSO Reductase Enzyme Family. Inorg. Chem..

[65] Lim B.S., Sung K.-M., Holm R.H. (2000). Structural and Functional Bis(dithiolene)-Molybdenum/Tungsten Active Site Analogues of the Dimethylsulfoxide Reductase Enzyme Family. J. Am. Chem. Soc..

[66] Lim B.S., Holm R.H. (2001). Bis(Dithiolene)molybdenum Analogues Relevant to the DMSO Reductase Enzyme Family: Synthesis, Structures, and Oxygen Atom Transfer Reactions and Kinetics. J. Am. Chem. Soc..

[67] Wang J.J., Kryatova O.P., Rybak-Akimova E.V., Holm R.H. (2004). Comparative Kinetics and Mechanism of Oxygen and Sulfur Atom Transfer Reactions Mediated by Bis(dithiolene) Complexes of Molybdenum and Tungsten. Inorg. Chem..

[68] Xiao Z., Bruck M.A., Enemark J., Young C.G., Wedd A.G. (1996). A Catalytic Cycle Related to Molybdenum Enzymes Containing [Mo VI O2 ]2+ Oxidized Active Sites. Inorg. Chem..

[69] Seo J., Shearer J., Williard P.G., Kim E. (2019). Reactivity of a biomimetic W (iv) bis-dithiolene complex with CO_2_ leading to formate production and structural rearrangement. Dalton Trans..

[70] Klimmek O., Kreis V., Klein C., Simon J., Wittershagen A., Kröger A. (1998). The function of the periplasmic Sud protein in polysulfide respiration of Wolinella succinogenes. Eur. J. Biochem..

[71] Dietrich W., Klimmek O. (2002). The function of methyl-menaquinone-6 and polysulfide reductase membrane anchor (PsrC) in polysulfide respiration of Wolinella succinogenes. Eur. J. Biochem..

[72] Nagarajan K., Joshi H.K., Chaudhury P.K., Pal K., Cooney J.J., Enemark J., Sarkar S. (2004). Structural and Functional Analogue of the Active Site of Polysulfide Reductase from Wolinella succinogenes. Inorg. Chem..

[73] Sugimoto H., Tajima R., Sakurai T., Ohi H., Miyake H., Itoh S., Tsukube H. (2006). Reversible sulfurization-desulfurization of tungsten bis(dithiolene) complexes. Angew. Chem. Int. Ed..

[74] Sugimoto H., Tano H., Toyota K., Tajima R., Miyake H., Takahashi I., Hirota S., Itoh S. (2010). Reduction of Bis(dithiolene)oxo(disulfido)tungsten(VI) Complex with Dihydrogen Related to the Chemical Function of the Fourth Tungsten-Containing Enzyme (WOR4) from Pyrococcus furiosus. J. Am. Chem. Soc..

[75] Dobbek H., Gremer L., Kiefersauer R., Huber R., Meyer O. (2002). Catalysis at a dinuclear [CuSMo(AO)OH] cluster in aCO dehydrogenase resolved at 1.1-Å resolution. Proc. Natl. Acad. Sci. USA.

[76] Fogeron T., Retailleau P., Chamoreau L.M., Li Y., Fontecave M. (2018). Pyranopterin Related Dithiolene Molybdenum Complexes as Homogeneous Catalysts for CO2 Photoreduction. Angew. Chem. Int. Ed..

[77] Fogeron T., Retailleau P., Chamoreau L.-M., Fontecave M., Li Y. (2017). The unusual ring scission of a quinoxaline-pyran-fused dithiolene system related to molybdopterin. Dalton Trans..

[78] Porcher J.-P., Fogeron T., Gomez-Mingot M., Derat E., Chamoreau L.-M., Li Y., Fontecave M. (2015). A Bioinspired Molybdenum Complex as a Catalyst for the Photo- and Electroreduction of Protons. Angew. Chem. Int. Ed..

[79] Fogeron T., Retailleau P., Gomez-Mingot M., Li Y., Fontecave M. (2018). Nickel Complexes Based on Molybdopterin-like Dithiolenes: Catalysts for CO2 Electroreduction. Organometallics.

[80] Fogeron T., Todorova T.K., Porcher J.-P., Gomez-Mingot M., Chamoreau L.-M., Mellot-Draznieks C., Li Y., Fontecave M. (2018). A Bioinspired Nickel(bis-dithiolene) Complex as a Homogeneous Catalyst for Carbon Dioxide Electroreduction. ACS Catal..

[81] Bradshaw B., Dinsmore A., Garner C.D., Joule J.A. (1998). Synthesis of a cobalt complex of a pyrano[2,3-b]quinoxaline-3,4-dithiolate related to molybdopterin. Chem. Commun..

[82] Bradshaw B., Collison D., Garner C.D., Joule J.A. (2001). Stable pyrano[2,3-b]quinoxalines and pyrano[2,3-g]pteridines related to molybdopterin. Chem. Commun..

[83] Bradshaw B., Dinsmore A., Ajana W., Collison D., Garner C.D., Joule J.A. (2001). Synthesis of the organic ligand of the molybdenum cofactor, in protected form. J. Chem. Soc. Perkin Trans. 1.

[84] Mouchfiq A., Todorova T.K., Dey S., Fontecave M., Mougel V. (2020). A bioinspired molybdenum–copper molecular catalyst for CO2 electroreduction. Chem. Sci..

[85] Appel A.M., Bercaw J.E., Bocarsly A.B., Dobbek H., DuBois D.L., Dupuis M., Ferry J.G., Fujita E., Hille R., Kenis P.J.A. (2013). Frontiers, Opportunities, and Challenges in Biochemical and Chemical Catalysis of CO_2_ Fixation. Chem. Rev..

[86] Takuma M., Ohki Y., Tatsumi K. (2005). Sulfido-Bridged Dinuclear Molybdenum−Copper Complexes Related to the Active Site of CO Dehydrogenase: [(dithiolate)Mo(O)S_2_ Cu(SAr)]^2−^ (dithiolate) 1,2-S_2_C_6_H_4_, 1,2-S_2_C_6_H_2_-3,6-Cl_2_, 1,2-S_2_C_2_H_4_). Inorg. Chem..

[87] Groysman S., Majumdar A., Zheng S.-L., Holm R.H. (2010). Reactions of Monodithiolene Tungsten(VI) Sulfido Complexes with Copper(I) in Relation to the Structure of the Active Site of Carbon Monoxide Dehydrogenase. Inorg. Chem..

[88] Gourlay C., Nielsen D.J., White J.M., Knottenbelt S.Z., Kirk M.L., Young C.G. (2006). Paramagnetic Active Site Models for the Molybdenum-Copper Carbon Monoxide Dehydrogenase. J. Am. Chem. Soc..

[89] Gourlay C., Nielsen D.J., Evans D.J., White J.M., Young C.G. (2018). Models for aerobic carbon monoxide dehydrogenase: Synthesis, characterization and reactivity of paramagnetic Mo(V)O(mu-S)Cu(I) complexes. Chem. Sci.

[90] Hollingsworth T.S., Hollingsworth R.L., Lord R.L., Groysman S. (2018). Cooperative bimetallic reactivity of a heterodinuclear molybdenum-copper model of Mo-Cu CODH. Dalton Trans..

[91] Dupé A., Hossain K., Schachner J.A., Belaj F., Lehtonen A., Nordlander E., Mösch-Zanetti N.C. (2015). Dioxomolybdenum(VI) and -tungsten(VI) Complexes with Multidentate Aminobisphenol Ligands as Catalysts for Olefin Epoxidation. Eur. J. Inorg. Chem..

[92] Dupé A., Judmaier M.E., Belaj F., Zangger K., Mösch-Zanetti N.C. (2015). Activation of molecular oxygen by a molybdenum complex for catalytic oxidation. Dalton Trans..

[93] Zwettler N., Ehweiner M.A., Schachner J.A., Dupé A., Belaj F., Mösch-Zanetti N.C. (2019). Dioxygen Activation with Molybdenum Complexes Bearing Amide-Functionalized Iminophenolate Ligands. Molecules.

[94] Schachner J.A., Belaj F., Mösch-Zanetti N.C. (2020). Isomers in chlorido and alkoxido-substituted oxidorhenium(V) complexes: Effects on catalytic epoxidation activity. Dalton Trans..

[95] Majumder S., Pasayat S., Roy S., Dash S.P., Dhaka S., Maurya M.R., Reichelt M., Reuter H., Brzezinski K., Dinda R. (2018). Dioxidomolybdenum(VI) complexes bearing sterically constrained aroylazine ligands: Synthesis, structural investigation and catalytic evaluation. Inorg. Chim. Acta.

[96] Maurya M.R., Dhaka S., Avecilla F. (2014). Synthesis, characterization and catalytic activity of dioxidomolybdenum(VI) complexes of tribasic pentadentate ligands. Polyhedron.

[97] Moradi-Shoeili Z., Boghaei D.M., Amini M., Bagherzadeh M., Notash B. (2013). New molybdenum(VI) complex with ONS-donor thiosemicarbazone ligand: Preparation, structural characterization, and catalytic applications in olefin epoxidation. Inorg. Chem. Commun..

[98] Trofimenko S. (1967). Boron-Pyrazole Chemistry. II. Poly(1-pyrazolyl)borates. J. Am. Chem. Soc..

[99] Trofimenko S. (1967). Transition Metal Poly(l-pyrazolyl)borates Containing Other Ligands. J. Am. Chem. Soc..

[100] Trofimenko S. (1967). Boron-pyrazole chemistry. IV. Carbon- and boron-substituted poly[(1-pyrazolyl) borates]. J. Am. Chem. Soc..

[101] Trofimenko S. (1969). Transition Metal Polypyrazolylborates Containing Other Ligands. J. Am. Chem. Soc..

[102] Trofimenko S., Calabrese J.C., Domaille P.J., Thompson J.S. (1989). Steric Effects in Polypyrazolylborate Ligands. Poly(3-isopropylpyrazolyl)borates: Ligands of Intermediate Steric Requirements. Inorg. Chem..

[103] Young C.G. (2016). Scorpionate Complexes as Models for Molybdenum Enzymes. Eur. J. Inorg. Chem..

[104] Xiao Z., Young C.G., Enemark J., Wedd A.G. (1992). A Single Model Displaying All the Important Centers and Processes Involved in Catalysis by Molybdoenzymes Containing [MoVI02]2+ Active Sites. J. Am. Chem. Soc..

[105] Paul T., Rodehutskors P.M., Schmidt J., Burzlaff N. (2016). Oxygen Atom Transfer Catalysis with Homogenous and Polymer-Supported N,N- and N,N,O-Heteroscorpionate Dioxidomolybdenum(VI) Complexes. Eur. J. Inorg. Chem..

[106] Maurya M.R. (2012). Catalytic Applications of Polymer-Supported Molybdenum Complexes in Organic Transformations. Curr. Org. Chem..

[107] Heinze K., Fischer A. (2007). Polymer-Supported Dioxido-MoVI Complexes as Truly Functional Molybdenum Oxotransferase Model Systems. Eur. J. Inorg. Chem..

[108] Emmanuel O., Orio M., Giorgi M., Réglier M., Iranzo O., Hardré R. (2018). An Air-Stable Molybdenum-Based Precatalyst in Oxygen-Atom Transfer Reactions. Eur. J. Inorg. Chem..

[109] Schachner J.A., Wiedemaier F., Zwettler N., Peschel L.M., Boese A.D., Belaj F., Mösch-Zanetti N.C. (2021). Catalytic reduction of nitrate by an oxidorhenium (V) complex. J. Catal..

[110] Harlan E.W., Berg J.M., Holm R.H. (1986). Thermodynamic Fitness of Molybdenum(IV,VI) Complexes for Oxygen Atom Transfer Reactions, Including Those with Enzymatic Substrates. J. Am. Chem. Soc..

[111] Yu S.-b., Holm R.H. (1989). Aspects of the Oxygen Atom Transfer Chemistry of Tungsten. Inorg. Chem..

[112] Lee S., Staley D., Rheingold A.L., Cooper N.J. (1990). Evidence for Photodisproportionation of d’-d1 Dimers [(MO{S2CN(CH2Ph)2}2)20] (M = molybdenum, tungsten) Containing Linear Oxo Bridges and for Oxygen Atom Transfer from [W02{S2CN(CH2Ph)2}2] to PEt3. Inorg. Chem..

[113] Eagle A.A., Tiekink E.R., Young C.G. (1997). Dioxotungsten(VI) Complexes of Hydrotris(3,5-dimethylpyrazol-1-yl)borate Including the X-ray Crystal Structure of the Tungsten Selenophenolate Complex cis-{HB(Me_2_C_3_N_2_H)_3_}WO_2_ (SePh). Inorg. Chem..

[114] Kaul B.B., Enemark J., Merbs S.L., Spence J.T. (1985). Molybdenum(VI)-Dioxo, Molybdenum(V)-Oxo, and Molybdenum(IV)-Oxo Complexes with 2,3:8,9-Dibenzo-l,4,7,10-tetrathiadecane. Models for the Molybdenum Binding Site of the Molybdenum Cofactor. J. Am. Chem. Soc..

[115] Sauer A., Kapelski A., Fliedel C., Dagorne S., Kol M., Okuda J. (2013). Structurally well-defined group 4 metal complexes as initiators for the ring-opening polymerization of lactide monomers. Dalton Trans..

[116] Sousa S.C.A., Fernandes A.C. (2015). Efficient deoxygenation methodologies catalyzed by oxo-molybdenum and oxo-rhenium complexes. Coord. Chem. Rev..

[117] Wong Y.-L., Yan Y., Chan E.S.H., Yang Q., Mak T.C.W., Ng K.P. (1998). cis-Dioxo-tungsten(VI) and -molybdenum(VI) complexes with N2O2 tetradentate ligands: Synthesis, structure, electrochemistry and oxo-transfer properties. J. Chem. Soc. Dalton Trans..

[118] Wong Y.-L., Yang Q., Zhou Z.-Y., Lee H.K., Mak T.C.W., Ng K.P. (2001). Synthesis, structure and oxo-transfer properties of dioxotungsten(VI) complexes with pyridine-based NO- and NS-bidentate ligands. New J. Chem..

[119] Lehtonen A., Sillanpää R. (2005). Dioxomolybdenum(VI) complexes with tri- and tetradentate aminobis(phenolate)s. Polyhedron.

[120] Maurya M.R., Mengesha B., Uprety B., Jangra N., Tomar R., Avecilla F. (2018). Oxygen atom transfer between DMSO and benzoin catalyzed by cis-dioxidomolybdenum(VI) complexes of tetradentate Mannich bases. New J. Chem..

[121] Hille R. (2013). The molybdenum oxotransferases and related enzymes. Dalton Trans..

[122] Sun S.-Q., Chen S.-L. (2019). How does Mo-dependent perchlorate reductase work in the decomposition of oxyanions?. Dalton Trans..

[123] Lehnert N., Musselmann B.W., Seefeldt L.C. (2021). Grand challenges in the nitrogen cycle. Chem. Soc. Rev..

[124] Kraft B., Strous M., Tegetmeyer H.E. (2011). Microbial nitrate respiration–Genes, enzymes and environmental distribution. J. Biotechnol..

[125] Averill B.A. (1996). Dissimilatory Nitrite and Nitric Oxide Reductases. Chem. Rev..

[126] Motzer W.E. (2001). Perchlorate: Problems, Detection, and Solutions. Environ. Forensics.

[127] Srinivasan A., Viraraghavan T. (2009). Perchlorate: Health Effects and Technologies for Its Removal from Water Resources. Int. J. Environ. Res. Public Health.

[128] Gulis G., Czompolyova M., Cerhan J.R. (2002). An Ecologic Study of Nitrate in Municipal Drinking Water and Cancer Incidence in Trnava District, Slovakia. Environ. Res..

[129] Ward M.H., Kilfoy B.A., Weyer P.J., Anderson K.E., Folsom A.R., Cerhan J.R. (2010). Nitrate Intake and the Risk of Thyroid Cancer and Thyroid Disease. Epidemiology.

[130] Abu-Omar M.M., McPherson L.D., Arias J., Béreau V.M. (2000). Clean and Efficient Catalytic Reduction of Perchlorate. Angew. Chem. Int. Ed..

[131] Abu-Omar M.M., Appelman E.H., Espenson J.H. (1996). Oxygen-Transfer Reactions of Methylrhenium Oxides. Inorg. Chem..

[132] Ford C.L., Park Y.J., Matson E.M., Gordon Z., Fout A.R. (2016). A bioinspired iron catalyst for nitrate and perchlorate reduction. Science.

[133] Drummond M.J., Miller T.J., Ford C.L., Fout A.R. (2020). Catalytic Perchlorate Reduction Using Iron: Mechanistic Insights and Improved Catalyst Turnover. ACS Catal..

[134] Elrod L.T., Kim E. (2018). Lewis Acid Assisted Nitrate Reduction with Biomimetic Molybdenum Oxotransferase Complex. Inorg. Chem..

[135] Ehweiner M.A., Wiedemaier F., Lajin B., Schachner J.A., Belaj F., Goessler W., Mösch-Zanetti N.C. (2021). Nature-Inspired Homogeneous Catalytic Perchlorate Reduction Using Molybdenum Complexes. ACS Catal..

[136] Ren C., Yang P., Gao J., Huo X., Min X., Bi E.Y., Liu Y., Wang Y., Zhu M., Liu J. (2020). Catalytic Reduction of Aqueous Chlorate With MoOx Immobilized on Pd/C. ACS Catal..

[137] Ren C., Yang P., Sun J., Bi E.Y., Gao J., Palmer J., Zhu M., Wu Y., Liu J. (2021). A Bioinspired Molybdenum Catalyst for Aqueous Perchlorate Reduction. J. Am. Chem. Soc..

[138] Ren C., Bi E.Y., Gao J., Liu J. (2022). Molybdenum-Catalyzed Perchlorate Reduction: Robustness, Challenges, and Solutions. ACS EST Eng..

[139] Lajin B., Goessler W. (2020). HPLC-ICPMS/MS shows a significant advantage over HPLC-ICPMS for the determination of perchlorate in ground, tap, and river water. Anal. Chim. Acta.

[140] Antony S., Bayse C.A. (2009). Theoretical Studies of Models of the Active Site of the Tungstoenzyme Acetylene Hydratase. Organometallics.

[141] Liao R.-Z., Yu J.-G., Himo F. (2010). Mechanism of tungsten-dependent acetylene hydratase from quantum chemical calculations. Proc. Natl. Acad. Sci. USA.

[142] Vincent M.A., Hillier I.H., Periyasamy G., Burton N.A. (2010). A DFT study of the possible role of vinylidene and carbene intermediates in the mechanism of the enzyme acetylene hydratase. Dalton Trans..

[143] Seiffert G.B., Ullmann G.M., Messerschmidt A., Schink B., Kroneck P.M.H., Einsle O. (2007). Structure of the non-redox-active tungsten/[4Fe:4S] enzyme acetylene hydratase. Proc. Natl. Acad. Sci. USA.

[144] Templeton J.L., Ward B.C., Chen G.J.-J., McDonald J.W. (1981). Oxotungsten(IV)-Acetylene Complexes: Synthesis via Intermetal Oxygen Atom Transfer and Nuclear Magnetic Resonance Studies. Inorg. Chem..

[145] Crane T.W., White P.S., Templeton J.L. (1999). Conversion of Tungsten(IV) Oxo−Alkyne Complexes to Oxo−Vinylidene Complexes. Organometallics.

[146] Peschel L.M., Belaj F., Mösch-Zanetti N.C. (2015). Towards Structural-Functional Mimics of Acetylene Hydratase: Reversible Activation of Acetylene using a Biomimetic Tungsten Complex. Angew. Chem. Int. Ed..

[147] Vidovic C., Peschel L.M., Buchsteiner M., Belaj F., Mösch-Zanetti N.C. (2019). Structural Mimics of Acetylene Hydratase: Tungsten Complexes Capable of Intramolecular Nucleophilic Attack on Acetylene. Chem. Eur. J..

[148] Yadav J., Das S.K., Sarkar S. (1997). A Functional Mimic of the New Class of Tungstoenzyme, Acetylene Hydratase. J. Am. Chem. Soc..

[149] Liu Y.-F., Liao R.-Z., Ding W.-J., Yu J.-G., Liu R.-Z. (2011). Theoretical investigation of the first-shell mechanism of acetylene hydration catalyzed by a biomimetic tungsten complex. J. Biol. Inorg. Chem..

[150] Schreyer M., Hintermann L. (2017). Is the tungsten(IV) complex (NEt4)2[WO(mnt)2] a functional analogue of acetylene hydratase?. Beilstein J. Org. Chem..

[151] Bondi R., Corovic M.Z., Buchsteiner M., Vidovic C., Belaj F., Mösch-Zanetti N.C. (2021). The Effect of Pyridine-2-thiolate Ligands on the Reactivity of Tungsten Complexes toward Oxidation and Acetylene Insertion. Organometallics.

[152] Ehweiner M.A., Belaj F., Kirchner K., Mösch-Zanetti N.C. (2021). Synthesis and Reactivity of a Bioinspired Molybdenum(IV) Acetylene Complex. Organometallics.

[153] Ehweiner M.A., Peschel L.M., Stix N., Corovic M.Z., Belaj F., Mösch-Zanetti N.C. (2021). Bioinspired Nucleophilic Attack on a Tungsten-Bound Acetylene: Formation of Cationic Carbyne and Alkenyl Complexes. Inorg. Chem..

[154] Clark R.E.D. (1936). The detection and colorimetric determination of tin by means of substituted 1:2-dimercaptobenzenes. A specific reagent for tin. Analyst.

[155] Mills W.H., Clark R.E.D. (1936). Stereochemistry of some new complex thio-salts of mercury, cadmium, and zinc. J. Chem. Soc..

[156] Clark R.E.D. (1937). The Colorimetric Determination of Tin by Means of Toluene-3:4-dithiol (“Dithiol”). Analyst.

[157] Gray H.B., Williams R., Bernal I., Billid E. (1962). A Spin-free Square Planar Cobaltous Complex. J. Am. Chem. Soc..

[158] Schrauzer G.N., Mayweg V. (1962). Reaction of Diphenylacetylene with Nickel Sulfides. J. Am. Chem. Soc..

[159] Davison A., Edelstein N., Holm R.H., Maki A.H. (1963). E.s.r. Studies of Four-Coördinate Complexes of Nickel, Palladium and Platinum Related by Electron Transfer Reactions. J. Am. Chem. Soc..

[160] Davison A., Edelstein N., Holm R.H., Maki A.H. (1963). The Preparation and Characterization of Four- Coordinate Complexes Related by Electron-Transfer Reactions. Inorg. Chem..

[161] Kramer S.P., Johnson J.L., Ribeiro A.A., Millington D.S., Rajagopalan K.V. (1987). The structure of the molybdenum cofactor. Characterization of di-(carboxamidomethyl)molybdopterin from sulfite oxidase and xanthine oxidase. J. Biol. Chem..

[162] Rajagopalan K.V., Johnson J.L. (1992). The Pterin Molybdenum Cofactors. J. Biol. Chem..

[163] Tanaka H., Okano Y., Kobayashi H., Suzuki W., Kobayashi A. (2001). A Three-Dimensional Synthetic Metallic Crystal Composed of Single-Component Molecules. Science.

[164] Coomber A.T., Beljonne D., Friend R.H., Brédas J.L., Charlton A., Robertson N., Underbill A.E., Kurmoo M., Day P. (1996). Intermolecular interactions in the molecular ferromagnetic NH4Ni(mnt)2· H_2_O. Nature.

[165] Wan W.L., Ji W., Zuo J.L., Bai J.F., You X.Z., Lim J.H., Yang S., Hagan D.J., Van Stryland E.W. (2000). Optical-limiting properties of neutral nickel dithiolenes. Appl. Phys. B.

[166] Liu H., Li X., Shi C., Wang D., Chen L., He Y., Zhao J. (2018). First-principles prediction of two-dimensional metal bis(dithiolene) complexes as promising gas sensors. Phys. Chem. Chem. Phys..

[167] Chakravarty C., Mandal B., Sarkar P. (2016). Bis(dithioline)-Based Metal–Organic Frameworks with Superior Electronic and Magnetic Properties: Spin Frustration to Spintronics and Gas Sensing. J. Phys. Chem. C.

[168] Kobayashi Y., Jacobs B., Allendorf M.D., Long J.R. (2010). Conductivity, Doping, and Redox Chemistry of a Microporous Dithiolene-Based Metal−Organic Framework. Chem. Mater..

[169] Pitchaimani J., Ni S.-F., Dang L. (2020). Metal Dithiolene Complexes in Olefin Addition and Purification, Small Molecule Adsorption, H2 Evolution and CO2 Reduction. Coord. Chem. Rev..

[170] Gomez-Mingot M., Porcher J.-P., Todorova T.K., Fogeron T., Mellot-Draznieks C., Li Y., Fontecave M. (2015). Bioinspired Tungsten Dithiolene Catalysts for Hydrogen Evolution: A Combined Electrochemical, Photochemical, and Computational Study. J. Phys. Chem. B.

[171] Porcher J.-P., Fogeron T., Gomez-Mingot M., Chamoreau L.M., Li Y., Fontecave M. (2016). Synthesis and Reactivity of a Bio-inspired Dithiolene Ligand and its Mo Oxo Complex. Chem. Eur. J..

[172] Fogeron T., Porcher J.-P., Gomez-Mingot M., Todorova T.K., Chamoreau L.M., Mellot-Draznieks C., Li Y., Fontecave M. (2016). A cobalt complex with a bioinspired molybdopterin-like ligand: A catalyst for hydrogen evolution. Dalton Trans..

[173] Eckenhoff W.T., Brennessel W.W., Eisenberg R. (2014). Light-Driven Hydrogen Production from Aqueous Protons using Molybdenum Catalysts. Inorg. Chem..

[174] Seo J., Kim E. (2012). O-Atom Exchange between H2O and CO2 Mediated by a Bis(dithiolene)tungsten Complex. Inorg. Chem..

[175] Harrison D.J., Lough A.J., Nguyen N., Fekl U. (2007). Push–Pull Molybdenum Trisdithiolenes Allow Rapid NonconventionalBinding of Ethylene at Ligand Sulfur Atoms. Angew. Chem. Int. Ed..

[176] Nguyen N., Lough A.J., Fekl U. (2012). Rapid, Covalent Addition of Phosphine to Dithiolene in a Molybdenum Tris(dithiolene). A New Structural Model for Dimethyl Sulfoxide Reductase. Inorg. Chem..

[177] Alvarez M., Galindo A., Pérez P.J., Carmona E. (2019). Molybdenum and tungsten complexes with carbon dioxide and ethylene ligands. Chem. Sci..

[178] Wang K., Stiefel E.I. (2001). Toward Separation and Purification of Olefins Using Dithiolene Complexes: An Electrochemical Approach. Science.

[179] Schrauzer G.N., Mayweg V. (1965). Preparation, Reactions, and Structure of Bisdithio-a-diketone Complexes of Nickel, Palladium, and Platinum’. J. Am. Chem. Soc..

[180] Schmitt R.D., Wing R.M., Maki A.H. (1969). Donor-Acceptor Complexes of the Inorganic pi Acceptor, Bis-cis- (1,2-perfluoromethylethene-1,2-dithiolato) nickel. J. Am. Chem. Soc..

[181] Herman A., Wing R.M. (1973). The structure of the nickel bis(1,2-ethenedithiolate)/2,3-dimethylbutadiene cycloaddition reaction product. J. Organomet. Chem..

[182] Shibl M.F., Dang L., Raju R.K., Hall M.B., Brothers E.N. (2013). A mechanism for the addition of ethylene to nickel bis-dithiolene. Int. J. Quantum. Chem..

[183] Yang T.-L., Ni S.-F., Zhang P., Dang L. (2016). Ligand effect on the reactivity difference of Mo Tris(dithiolene) complexes towards Ethylene: A computational study. J. Organomet. Chem..

[184] Dang L., Shibl M.F., Yang X., Alak A., Harrison D.J., Fekl U., Brothers E.N., Hall M.B. (2012). The Mechanism of Alkene Addition to a Nickel Bis(dithiolene) Complex: The Role of the Reduced Metal Complex. J. Am. Chem. Soc..

[185] Dang L., Yang X., Zhou J., Brothers E.N., Hall M.B. (2012). Computational Studies on Ethylene Addition to Nickel Bis(dithiolene). J. Phys. Chem. A.

[186] Dang L., Ni S.-F., Hall M.B., Brothers E.N. (2014). Uptake of One and Two Molecules of 1,3-Butadiene by Platinum Bis(dithiolene): A Theoretical Study. Inorg. Chem..

[187] Tang Q., Zhou Z. (2013). Electronic Properties of π-Conjugated Nickel Bis(dithiolene) Network and Its Addition Reactivity with Ethylene. J. Phys. Chem. C.

